# A novel spontaneous model of epithelial-mesenchymal transition (EMT) using a primary prostate cancer derived cell line demonstrating distinct stem-like characteristics

**DOI:** 10.1038/srep40633

**Published:** 2017-01-17

**Authors:** Naomi Harner-Foreman, Jayakumar Vadakekolathu, Stéphanie A. Laversin, Morgan G. Mathieu, Stephen Reeder, A. Graham Pockley, Robert C. Rees, David J. Boocock

**Affiliations:** 1 John van Geest Cancer Research Centre, Nottingham Trent University, Nottingham, NG11 8NS, UK

## Abstract

Cells acquire the invasive and migratory properties necessary for the invasion-metastasis cascade and the establishment of aggressive, metastatic disease by reactivating a latent embryonic programme: epithelial-to-mesenchymal transition (EMT). Herein, we report the development of a new, spontaneous model of EMT which involves four phenotypically distinct clones derived from a primary tumour-derived human prostate cancer cell line (OPCT-1), and its use to explore relationships between EMT and the generation of cancer stem cells (CSCs) in prostate cancer. Expression of epithelial (E-cadherin) and mesenchymal markers (vimentin, fibronectin) revealed that two of the four clones were incapable of spontaneously activating EMT, whereas the others contained large populations of EMT-derived, vimentin-positive cells having spindle-like morphology. One of the two EMT-positive clones exhibited aggressive and stem cell-like characteristics, whereas the other was non-aggressive and showed no stem cell phenotype. One of the two EMT-negative clones exhibited aggressive stem cell-like properties, whereas the other was the least aggressive of all clones. These findings demonstrate the existence of distinct, aggressive CSC-like populations in prostate cancer, but, importantly, that not all cells having a potential for EMT exhibit stem cell-like properties. This unique model can be used to further interrogate the biology of EMT in prostate cancer.

Prostate cancer is a major cause of morbidity and mortality in men, particularly in the developed world. Despite advances in detection and treatment methods, disease relapse is a common occurrence and progressive hormone refractory metastatic prostate cancer remains an incurable disease.

In recent years, the cancer stem cell (CSC) hypothesis has emerged as a compelling but controversial model for cancer progression^1^,^2^,^3^. In addition to tumour initiation, cancer stem cells are considered to be accountable for tumour differentiation, tumour maintenance, dissemination, drug resistance and relapse following therapy in various cancers^4^,^5^,^6^,^7^,^8^,^9^,^10^,^11^. Of late, there has been much evidence to suggest that cancer cells reactivate the latent embryonic programme known as epithelial to mesenchymal transition (EMT) in order to acquire the invasive and migratory properties that are necessary for the successful completion of the invasion-metastasis cascade^12^. Intriguingly, the EMT programme has been implicated in the generation of cells with the properties of stem cells in breast cancer models^13^,^14^. Since metastasis is accountable for the vast majority (~90%) of cancer-associated mortalities and CSCs are implicated in therapy failure and subsequent cancer relapse, it is apparent that EMT and CSCs are of utmost clinical relevance. An improved understanding of the events and processes concerning these phenomena is therefore likely to reveal new therapeutic opportunities for preventing and treating aggressive disease in many clinical settings.

As with many other solid cancer models, EMT is believed to play a critical role in the metastatic spread of prostate cancer^15^. *In vitro* and *in vivo* models of EMT in prostate cancer have provided insight into several mechanisms that are involved in EMT, of which androgen deprivation^16^ and TGF-β signalling^17^ are of particular clinical relevance. To date, the majority of observations concerning EMT in cancer have been derived from *in vitro* cell models, in which EMT is mainly induced by ectopic expression of EMT-inducing transcription factors or by stimulation with growth factors such as TGF-β^13^,^18^,^19^,^20^. Unlike many other cancers, the availability of cell lines that are derived from primary prostate tumours is limited^21^. Moreover, the standard cell lines for prostate cancer research, such as PC3, DU145 and LNCaP, are derived from metastatic rather than primary disease^22^. Understanding the invasive/migratory and tumour initiating properties in a cell line derived from primary tumour site may provide relevant information in the triggering of the initial metastatic cascade. In this study, we therefore examined the less commonly used, primary tumour-derived cell lines: OPCT-1, OPCT-2, P4E6, in addition to the commercially available, metastasis-derived PC-3 and DU145 cell lines, for evidence of spontaneous EMT events in normal culture conditions. We then derived and interrogated phenotypically distinct, stable clonal OPCT-1 progenies with differential features of EMT potential.

## Results

**Figure 1:**
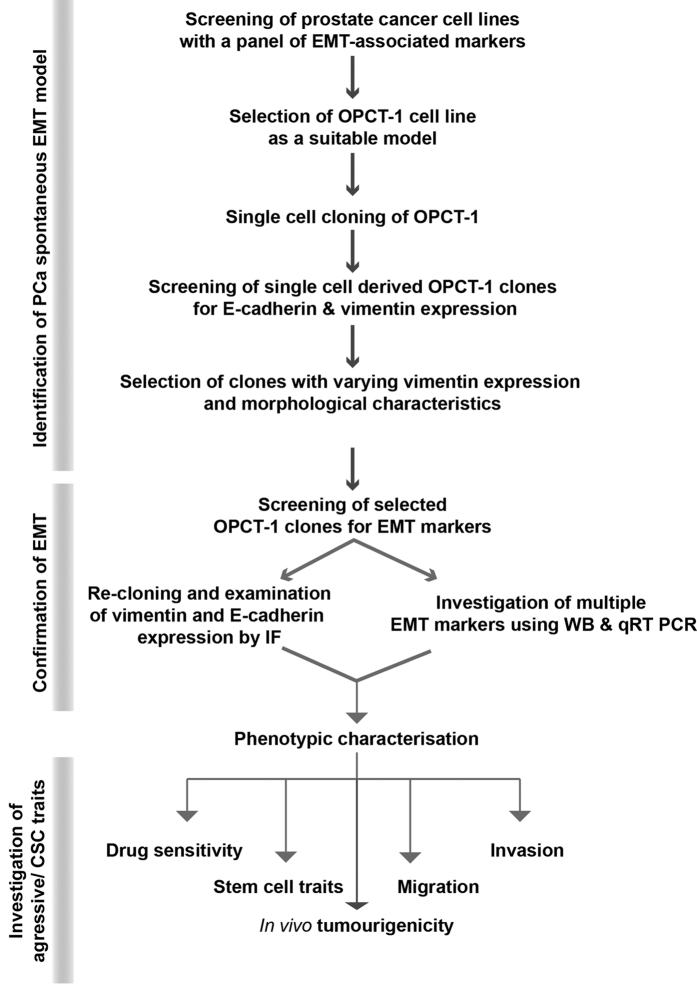
Flow chart demonstrating the steps involved in the identification of a prostate cancer cell line with non-exogenously induced EMT events, followed by the generation and interrogation of a model to investigate the relationship between EMT and CSCs in human prostate cancer.

A summary of the processes involved in the identification, interrogation and generation of a spontaneous human prostate cancer EMT model is given in Fig. 1.

### OPCT-1 is an appropriate cell line for the investigation of EMT in human prostate cancer

**Figure 2:**
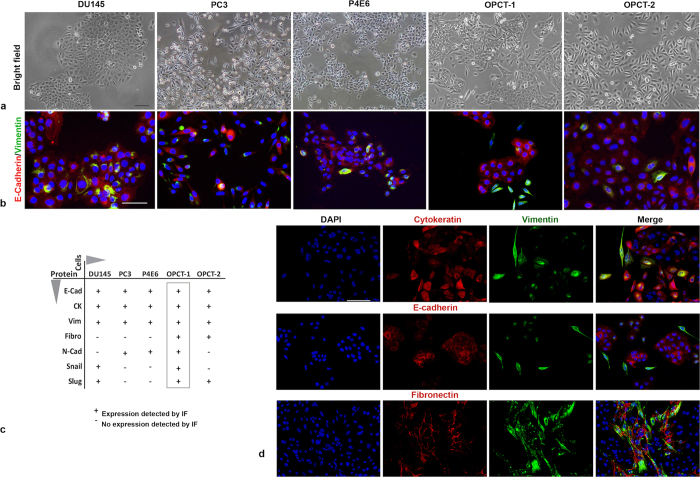
Identification of OPCT-1 as a suitable model for the study of spontaneous EMT in prostate cancer. (**a**) Bright field images of human prostate cancer cell lines derived from metastatic lesions: DU145 and PC3, and primary tissues: P4E6, OPCT-1 and OPCT-2 (Image magnification at x10). (**b**) Dual immunofluorescent staining of DU145, PC3, P4E6, OPCT-1 and OPCT-2 using antibodies against E-cadherin (red) and vimentin (green) (*n* = 3). (**c**) Table summarising the results of the IF screening of DU145, PC3, P4E6, OPCT-1 and OPCT-2 cells for the expression of several EMT-associated markers. (**d**) Summary composite of OPCT-1 stained with common markers used to investigate EMT: Cytokeratin pan/vimentin, E-cadherin/vimentin, fibronectin/vimentin. Scale bar: 50 μM.

Five androgen-independent human prostate cancer cell lines, two derived from metastatic lesions (DU145, PC3) and three derived from primary tissues (P4E6, OPCT-1, OPCT-2), were selected for the purpose of this study. Upon microscopic examination, phenotypic differences in cellular morphology (i.e. cobblestone vs fibroblastoid), were apparent (Fig. 2a). We therefore speculated that the cell lines might exhibit distinct patterns of epithelial and/or mesenchymal protein expression. To test this possibility, we examined the expression of several EMT-associated markers (E-cadherin, vimentin, cytokeratin, fibronectin, N-cadherin, Snail and Slug), by immunofluorescence.

Widely used to identify cells of epithelial origin, E-cadherin is a key component in the formation of cell-cell adherens-type junctions in epithelial tissues. Although typically expressed between cells at the cell surface, during the development of most epithelial cancers E-cadherin-mediated cell-cell adhesion is lost and changes in expression from the membrane to the cytoplasm are often observed^23^,^24^,^25^. Vimentin is an intermediate filament protein which is ubiquitously expressed by mesenchymal cells, as such, it is the most commonly used marker for identifying cells of mesenchymal origin^26^. Immunofluorescent staining with E-cadherin and vimentin antibodies revealed that all five cell lines expressed E-cadherin. However, the staining intensity, distribution and frequency of expression of vimentin varied markedly across the cell lines (Fig. 2b). Metastatic disease-derived cell lines, PC3 and DU145, demonstrated focal vimentin expression, as did P4E6 cells (Fig. 2b). In contrast, the primary tumour-derived cell lines, OPCT-1 and OPCT-2, exhibited differential non-focal expression of vimentin, with sophisticated networks of vimentin fibres throughout some cells and lower levels of vimentin appearing around the nucleus in other cells (Fig. 2b). Of the five prostate cancer cell lines examined, only OPCT-1 and OPCT-2 contained cells expressing the mesenchymal marker fibronectin (Supplementary Figure 1; summarised in Fig. 2c). Regarding OPCT-1, fibronectin was predominantly co-expressed with vimentin (Fig. 2d). Conversely, fibronectin expression was not confined to vimentin-positive cells in the OPCT-2 cell line (data not shown). The overall data obtained from this screening are summarised in Fig. 2c. Of the cell lines screened, OPCT-1 was comprised of the most distinct populations: E-cadherin-positive/vimentin-negative cells; which formed colonies, vimentin/fibronectin-positive, spindle-shaped cells; which were situated between the colonies and dual E-cadherin/vimentin-positive cells (Fig. 2d). Remarkably, the single vimentin-positive cells in this cell line grew in isolation and demonstrated fibroblastoid morphologies, a feature which is consistent with cells of mesenchymal origin. Furthermore, unlike DU145, PC3, P4E6 and OPCT-2, OPCT-1 demonstrated positive staining for all of the EMT-associated markers examined (Fig. 2c). To assess the characteristics of this cell line with regard to basal intermediate and luminal origin within the prostate, we have employed a quantitative real-time PCR based assay to determine the expression of markers that distinguish the hierarchy of prostate cancer cells (AR, PSA, cytokeratin 18, 14, 8, 5 and p63) alongside three widely used human prostate cancer cell lines - LNCAP, DU-145 and PC-3. We found that this cell line has an intermediary phenotype with high expression of cytokeratin-5 and low expression of CK14 and no detectable p63 (Supplementary Figure 7).

Based on these findings, the OPCT-1 cell line was selected as the basis for the derivation of clones that would be used for further investigation of EMT in human prostate cancer.

### Clonally-derived OPCT-1 cultures exhibit distinct EMT-associated protein expression patterns

To confirm that the vimentin-positive OPCT-1 cells had not arisen as a result of stromal contamination during the initial derivation of the cell line, and also to demonstrate that the OPCT-1 cell line contained a population of cells which were transitioning between epithelial and mesenchymal states, we performed a limiting dilution cloning assay. After 21 days, a total of 51 clones were obtained, expanded, cryopreserved at three passages, and then screened for the expression of E-cadherin and vimentin by immunofluorescence. Although high levels of E-cadherin were detected in all of the clones, a large variation in the number of vimentin-positive cells within each clone was evident, and vimentin was detected in all but two of the 51 clones examined (Supplementary Table 1). Expression of both E-cadherin and vimentin by clonally-derived populations of OPCT-1 cells confirmed that the vimentin-positive cells had not arisen as a result of stromal contamination. Furthermore, apparent morphological differences between vimentin-positive/E-cadherin negative and E-cadherin-positive/vimentin-negative cells in many of the clones suggested that they contained both epithelial and mesenchymal populations. Intriguingly, variation in the levels of vimentin expression across the clones revealed that individual OPCT-1 cells differed in their ability and frequency to transdifferentiate (Summarised in Supplementary Table 1).

**Figure 3:**
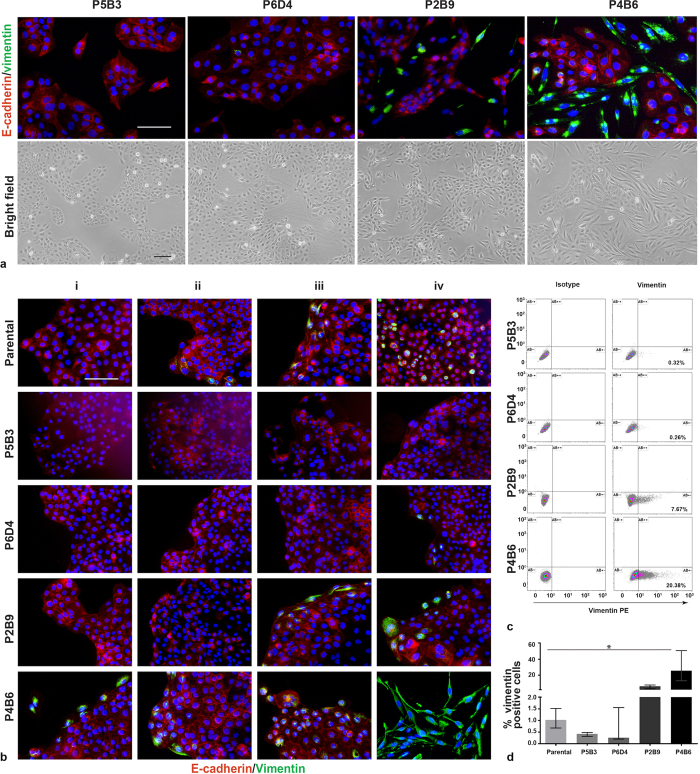
(**a**) Representative bright field images of four OPCT-1 clones of interest; P5B3, P6D4, P2B9 and P4B6 and their corresponding dual immunofluorescent (IF) staining profile using antibodies against E-cadherin (red) and vimentin (green). Nuclear staining (blue) was achieved using mounting media with DAPI. (*n* = 3). (**b**) Dual immunofluorescent staining of re-cloned clones P5B3, P6D4, P2B9, P4B6 and parental OPCT-1. Column i–iv are the representative images of separate wells from across the three assays. Scale bar: 50 μM. (**c**) Representative flow cytometric data of vimentin-positive cells present in each of the clones and parental OPCT-1, % of vimentin-positive cells are given in the bottom right quadrant. Intracellular staining of vimentin was achieved using mouse anti-human vimentin-PE and mouse IgG1k-PE isotype control antibody was used as a staining control. (**d**) Bar graph showing the percentage of vimentin-positive cells, each bar represents % median expression and the error bars represent the interquartile range. Significant differences were calculated, using the non-parametric Kruskal Wallis test (*p* = 0.0048; *Kruskal Wallis statistic* <14.94; *df* = 4.) (*n* = 4).

We subsequently shortlisted 12 clones of interest, all of which were screened for morphological and protein expression changes following successive freezing and passaging (Supplementary Figure 2). From these, four phenotypically stable and distinct clones were selected for further investigation. The clones were designated P5B3, P6D4, P2B9 and P4B6, and ranged from very low to high with regard to vimentin expression (Fig. 3a). All observations of the OPCT-1 clones were carried out within four passages. The phenotypes of the OPCT-1 clones, as observed by immunofluorescence, remained stable throughout the study: with P5B3 and P6D4 consistently exhibiting the smallest, and P2B9 and P4B6 repeatedly demonstrating the largest populations of vimentin-positive cells.

Prior to further characterisation of the four OPCT-1 clones of interest, we verified their ability to generate mixed E-cadherin/vimentin-positive populations from a single cell. The use of a cell sorter to seed single cells directly into fluorescence-compatible 96 well plates enabled minimal handling of the (re)cloned cells prior to conducting immunofluorescence, directly in the wells in which they had grown. This method therefore ensured that the presence of mixed cell populations could not have been due to contamination during cell culture. This assay confirmed that cells expressing both epithelial (E-cadherin) and mesenchymal (vimentin) markers arose in single-cell-derived populations of OPCT-1 clones (Fig. 3b, representative images). Though E-cadherin expression in the clonal progenies was present in the selected clones, the staining indicated non-membranous expression and therefore represented a non-functional E-cadherin. We therefore re-stained the clones using confocal microscopy and generated stacked images of 30–40 sections from each of the clones to ascertain their localisation. To quantitatively determine the membrane expressing E-cadherin cells, we scored three images (300 cells) from each of the clones and expressed the percentage of membrane E-cadherin expressing cells as a bar graph (Supplementary Figure 3). The data showed that all the clones except clone P2B9 had a significant proportion of the cells expressing membrane localised E-cadherin. Even though a number of non-membrane expressing cells were observed in clones P5B3, P6D4 and the Parental cell line, there were no significant differences observed. However, in clone P4B6, all the epithelial cells stained for strong membrane localised E-cadherin.

We quantitatively assessed the number of vimentin-positive cells that were present in each of the four clones and the parental cell line using flow cytometry (Fig. 3c, representative data, and D). Differences in vimentin expression between the clones were highly significant (p < 0.0048; Kruskal Wallis statistics = 14.94; df = 4). Moreover, these data supported the immunofluorescence-based observations and confirmed that the percentage of vimentin-positive cells in clones P5B3 (0.42%) and P6D4 (0.26%) was lower than that of parental OPCT-1 (1.02%), whereas clones P2B9 (6.22%) and P4B6 (25.69%) comprised a larger number of vimentin-positive cells (i.e. were enriched).

**Figure 4:**
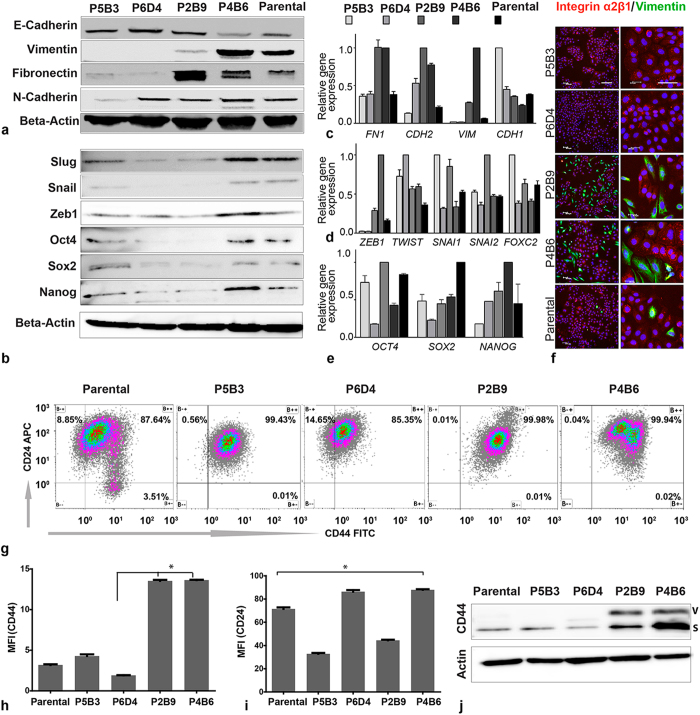
(**a**,**b**) Representative Western blot images of selected EMT and cancer stem cell associated marker expression by clones P5B3, P6D4, P2B9, P4B6 and parental OPCT-1 (*n* = 5). Beta-actin was used as a loading control in each set of the experiments. (**c**) Relative gene expression of common EMT associated marker genes fibronectin, E-cadherin, vimentin and N-cadherin (*n* = 3). (**d**) Quantitative gene expression analysis of common EMT transcription factors *ZEB1, TWIST, SNAI1, SNAI2* and *FOXC2*. (**e**) Quantitative gene expression analysis of embryonic stem cell genes *NANOG, OCT4* and *SOX2*. Real-time PCR values were normalised to the housekeeping gene HPRT (*n* = 3), expression of each gene was normalised to its highest expressing sample among the five genotypes studied. Each bar represents the mean of three independent experiments and the error bars represent the standard deviation. (**f**) Dual immunofluorescence staining of vimentin (green) and integrin α2β1 (red) in parental and all clonal progenies at two magnifications 10× (left column) and 20× (right column). Scale bar: 50 μM. (**g**) Dot plot showing the flow cytometry surface staining of CD44 and CD24 molecules on parental and clonal progenies of OPCT-1. (**h**) Staining intensity of CD44 assessed by mean fluorescence (*n* = *4*). (**i**) Staining intensity of CD24 assessed by mean fluorescence (n = 4). (**j**) Western blot image showing expression of CD44 v & s variants. Unprocessed original scans of the blots are shown in Supplementary Figure 9.

Real-time quantitative PCR and Western blot analyses further supported our observations regarding vimentin and E-cadherin expression (Fig. 4a and c). To further characterise the OPCT-1 clones, we investigated the expression of two additional EMT-associated markers (fibronectin and N-cadherin) by Western blotting, real time quantitative PCR and immunofluorescence (Fig. 4a and c and Supplementary Figure 4). The expression patterns of these mesenchymal proteins confirmed earlier observations that two of the clones (P5B3 and P6D4) were highly epithelial, whereas two (P2B9 and P4B6) contained mesenchymal populations. However, the expression of five EMT-associated transcription factors was not consistent with vimentin-positivity. qRT-PCR data revealed that the mRNA levels of the EMT-activator, *ZEB1* were highest in the most vimentin-positive clone (P4B6), followed by the second most vimentin positive clone (P2B9) (Fig. 4d). Clone P2B9 also demonstrated high levels of *SNAI1* and *SNAI2* expression. However, the vimentin-low clone P5B3 showed the highest *SNAI1* levels at the mRNA level (Fig. 4d). Interestingly, the mRNA expression signatures of the transcription factors *TWIST* and *FOXC2* did not correlate with other EMT-associated markers in this model (Fig. 4d). Consistent with observations which revealed the presence of mesenchymal populations in clone P4B6, transcription factors Zeb1, Snail and Slug were upregulated at the protein level in this clone (Fig. 4b and Supplementary Figure 4). However, this was not consistent with clone P2B9, the second most vimentin-positive clone (Fig. 4b). These data suggest that EMT events may be governed by distinct molecular mechanisms, even in clonal populations derived from the same primary tumour.

### Prostate cancer stem cells and EMT-derived mesenchymal cells are mutually exclusive

Having developed and characterised a model representing a range of epithelial and mesenchymal phenotypes, it was then employed to explore previously observed phenomena, in the context of EMT.

In 2005, Collins *et al*. identified putative prostate cancer stem cells using a panel of markers which had also been used to identify normal prostate stem cells^27^ and in 2008, Mani *et al*. revealed that EMT generates cells with the properties of cancer stem cells^13^. We therefore postulated that EMT of prostate cancer cells may give rise to cells with the properties of prostate stem cells. To address this, we examined the expression of three reported prostate cancer stem cells markers, CD44, CD24 and Integrin α2β1, by the OPCT-1 clones. In addition, we examined the expression of three stem cell-associated transcription factors that are known to play a key role in the maintenance of self-renewal and pluripotency: Nanog, Oct4 and Sox2. We predicted that these markers would be more highly expressed in clones P2B9 and P4B6, both of which contain mesenchymal populations.

CD44+ and CD24− cells have been reported in prostate cancer cell models as putative tumour initiating/cancer stem cells^28^,^29^,^30^. Interestingly, all the OPCT-1 clones expressed CD44, a family of proteins shown to regulate growth, survival, differentiation and migration of cancer cells^29^ (Supplementary Figure 5). The detection of CD44 was also carried out using Western blotting, flow cytometry and immunofluorescence. With the Western blot analysis, CD44 expression was detected in all clonal progenies and also the parental cell line. The highest expression of CD44 was observed in the clones P2B9 and P4B6, which which contain EMT-derived, vimentin-positive populations (Fig. 4j). Conversely, the lowest levels of CD44 were observed in clones P5B3, P6D4 and parental (Fig. 4j). Similar patterns of CD44 expression were observed by flow cytometry (Fig. 4g and h). Two isoforms of CD44 have been reported in many cancers, a commonly expressed CD44s and less commonly expressed CD44v. A variant switching of CD44v to CD44s has been reported in breast cancer cell lines with EMT^30^. Expression of CD44s was found to be markedly increased in both clones P2B9 and P4B6 (Clones with a higher incidence of EMT events). However, we also detected the CD44v isoform in these clones. CD44s expression was found to be a function of EMT events in these clones, with the highest expression in P4B6 (the clone having the highest percentage of EMT-derived mesenchymal cells) and the second highest in P2B9 (the clone having the next most EMT-derived mesenchymal cells) (Fig. 4j).

Expression of CD24 was also assessed using flow cytometry. CD24 showed a marked difference in surface expression between the clones, with the highest expression in clones P4B6 and P6D4, followed by the parental cell line (assessed by mean fluorescence). However, the surface expression of CD24 was found to be significantly lower in clones P5B3 and P2B9 (Fig. 4g and I). The co-expression pattern of CD44 and CD24 on the surface of the clonal progenies and the parental cell lines, showed that there are distinct variations in surface expression patterns, especially in the parental cell line, with a distinctive CD44+ CD24− population and this phenotype has been reported in several cancers as having a high tumour-initiating potential^29^,^30^.

Integrin α2β1 is a transmembrane receptor for extracellular matrix proteins (such as collagen and laminin), adhesion molecules (such as E-cadherin), and several other ligands (including matrix metalloproteinase-1). Among other functions, it is known to play a role in the generation and organisation of extracellular matrix proteins, and to mediate interactions between adhesion molecules on adjacent cells^31^. Dual immunofluorescent staining for vimentin and Integrin α2β1 revealed differential expression patterns (Fig. 4f). Since the EMT-derived, vimentin-positive cells failed to demonstrate Integrin α2β1 expression, these data revealed that EMT-derived prostate cancer cells and the prostate cancer stem cells identified by Collins *et al*.^27^ are mutually exclusive. Importantly, this experiment failed to support the hypothesis that EMT of prostate cancer cells gives rise to prostate cancer stem cells.

Oct4, Sox2 and Nanog are transcription factors that are responsible for the regulation and maintenance of pluripotency in embryonic stem cells^32^. Their similar role in epithelial cancers had been investigated in many studies^33^,^34^. To understand their regulation in a model with different EMT potential, we investigated the expression patterns of these transcription factors in the clones, at both the mRNA and protein level.

At the mRNA level, the highest expression of *NANOG* was detected in clones P4B6 and P2B9, both of which contained mesenchymal populations (Fig. 4e). However, *OCT4* and *SOX2* mRNA expression did not correlate with the vimentin positivity: *OCT4* expression was highest in clone P2B9, but low in clone P4B6, and *SOX2* expression was lower in both clones that were enriched for mesenchymal cells. Overall, these data revealed a correlation between *NANOG* expression and EMT characteristics, but failed to reveal the same correlation with *OCT4* and *SOX2*.

Western blot analysis demonstrated that all three of the stem cell-associated transcription factors were present at the protein level in clone P4B6 (Fig. 4b). These data suggest a correlation between EMT and pluripotency. However, the second most vimentin-positive clone (P2B9) showed very low protein expression of these transcription factors. Interestingly the most epithelial clone (P5B3) was also found to express the above transcription factors; thereby indicating that EMT events and the expression of pluripotency factors are unrelated in this model (Fig. 4b).

### Expression of EMT-associated markers does not necessarily correspond with aggressive stem-like behaviour

Having profiled the OPCT-1 clones with regard to expression of EMT and CSC-associated markers, we conducted *in vitro* and *in vivo* assays to determine whether the expression of EMT-associated markers corresponded with properties attributed to an aggressive, migratory, cancer stem cell-like phenotype.

**Figure 5:**
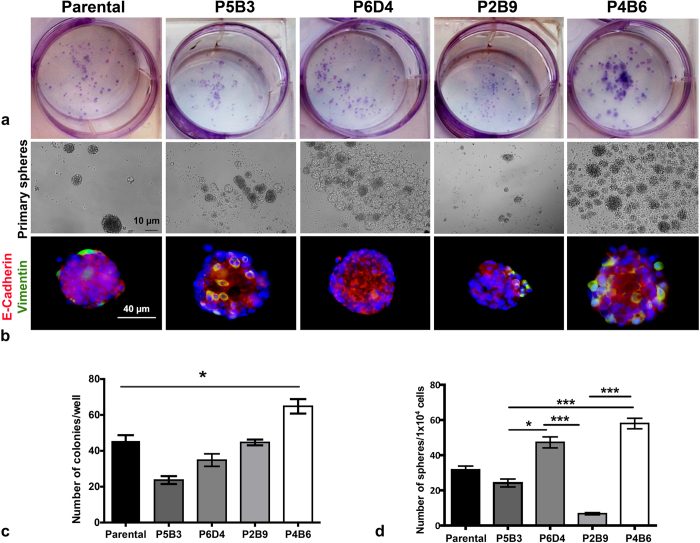
Assessment of stem cell characteristics of OPCT-1 clonal progenies. (**a**) Representative images of the colonies. Cells were plated at clonal density, cultured for a period of 10 days, fixed with ethanol and stained with crystal violet prior to enumerating the colonies. (**b**) Representative bright field and immunofluorescence images from the sphere-forming assay performed on clones P5B3, P6D4, P2B9, P4B6 and parental OPCT-1. Cells were plated in ultra-low adherent 24-well plates at clonal density in normal medium, and cultured over a period of 12 days (n = 3). Scale bar: 50 μM. (**c**) Bar graph showing the number of colonies obtained from parental and each of the OPCT-1 clones, each bar represent the mean ± SEM. Significant differences were calculated by the nonparametric Friedman test. (p = *0.0003, Friedman Statistic* < *21.01, df* = *4*). (**d**) Bar graph showing number of primary sphere-formation., each bar represents the mean of three assays and the error bars represent the SEM. Significant differences were calculated by the non-parametric Kruskal-Wallis test. (*p* = *0.0001, Kruskal-Wallis statistic* < *54.60, df* = *4*), Dunn’s multiple comparison test was used for pairwise comparisons. the number of cells seeded in each assay is indicated on the Y axis.

Cancer stem cells have been shown to demonstrate increased clonogenic capacity compared with other cancer cells, and clonogenic assays have been used to identify populations of prostate cancer cells with stem-like characteristics^35^. Herein, the clonogenicity of the four OPCT-1 clones and parental OPCT-1 cells was determined using an assay, wherein cells were seeded at low (clonal) density and allowed to grow prior to counting the number of colonies. The cells which formed the largest number of colonies were deemed the most “clonogenic”. Highly significant differences were observed in the clonogenic abilities of the clones (non-parametric Friedman ANOVA, (p < 0.0003, Friedman statistic < 21.01, df = 4) (Fig. 5a and c). Clone P4B6 and clone P5B3 possessed the highest and lowest clonogenicities, respectively. This assay confirmed that the two most vimentin-positive clones, clone P4B6 and clone P2B9, were also the most clonogenic. Hence, this assay appeared to demonstrate a correlation between EMT and clonogenicity. However, the colonies formed by these clones were very dissimilar; clone P2B9 formed small colonies comprised of few cells, whereas clone P4B6 formed large, diverse colonies that were comprised of many cells (Fig. 5a).

Sphere-forming assays are widely employed to assess stemness in cancer cell populations^36^. We therefore examined the sphere-forming capacity of each of the OPCT-1 clones and parental OPCT-1. Although all four clones were capable of anchorage-independent growth (Fig. 5 and d, Primary spheres), the sphere-forming abilities of the clones differed significantly (p < 0.0001, Kruskal-Wallis statistic < 54.60, df = 4). Our data revealed that clone P4B6 and clone P2B9 were the most and least sphere-forming populations, respectively. Although the clone which was most capable of forming spheres was also the most vimentin-positive (P4B6), the second most vimentin-positive clone (P2B9) was the least capable of sphere-formation in non-adherent conditions (Fig. 5b and d). Moreover, clones P5B3 and P6D4, which showed fewer EMT marker characteristics, formed more spheres than clone P2B9 (Fig. 5b and d). Therefore, EMT does not necessarily bestow enhanced sphere-forming ability.

As clone P2B9 was less capable of forming spheres than parental OPCT-1, this clone appeared to possess a smaller cancer stem/progenitor cell population than the parental cell line from which they were derived, despite being enriched for vimentin-positive, EMT-derived cells. In contrast, clones P5B3, P6D4 and P4B6 formed more spheres than parental OPCT-1.

We also used immunofluorescence to investigate the expression of E-cadherin and vimentin in the primary spheres (Fig. 5b). Interestingly, vimentin-positive cells were observed in all of the spheres, irrespective of the clones from which they were generated (Fig. 5b). Furthermore, vimentin-positive cells were present in both the centre and on the surface of the spheres. Surprisingly, clone P5B3 formed spheres with a relatively high number of vimentin-positive cells. This was unexpected, as this clone possessed a very low population of vimentin-positive cells in 2D culture conditions. Consistent with what was observed in 2D culture, clone P4B6 formed spheres with the largest population of vimentin-positive cells. Spheres formed by clone P6D4 demonstrated the highest levels of E-cadherin and the lowest levels of vimentin expression. Interestingly, E-cadherin expression was low on the perimeter of the spheres (Fig. 5b).

**Figure 6:**
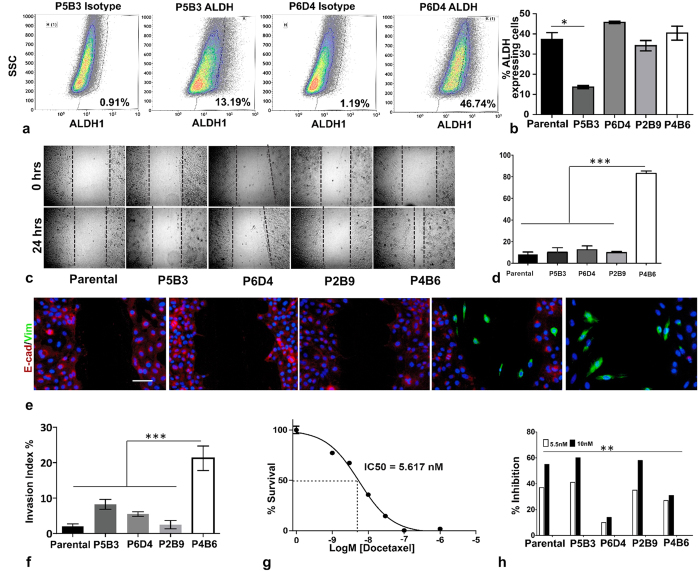
(**a**) The results of the Aldefluor assay, with representative flow cytometric data from which the percentage of ALDH1hi cells present in each of the clones and parental OPCT-1 was determined. Representative data showing the least and the most ALDH1 activity as a density dot-plot. Side scatter is represented on the Y-axis and ALDH1 staining is represented on the X-axis. Representative isotype control staining is also given (*n* = 3). (**b**) Percentge of ALDH1 high cells in parental and clonal progenies of OPCT-1. Data presented as median ± interquartile range. Significant differences were calculated by the nonparametric Kruskal-Wallis test (*p* = 0.0244*, Kruskal-Wallis statistic* = 12.89*, df* = 4 *n* = 4). (**c**) Representative images from the *in vitro* scratch assay showing wound closure after 24 h of wounding. (**d**) Percentage of wound closure after 24 h represented as bar graph. (**e**) Dual immunofluorescent staining was used to determine the phenotype of migratory cells, E-cadherin (red) and vimentin (green). Scale bar: 50 μM. (**f**) Results of the Matrigel invasion assay. Data are presented as the median ± interquartile range. Significant differences were calculated by the nonparametric Friedman test. (*p* = 0.0024*, Friedman statistic* = 18.49 *n* = 3). (**g**) Dose response curve of parental OPCT-1 cell line to docetaxel measured using the thymidine proliferation assay. The cell line was treated with a range of concentrations of docetaxel to reveal a dose-dependent growth response. The IC_50_ concentration of the drug was calculated using GraphPad Prism software (*n* = 3). The y-axis represents the normalised drug response, the x-axis represents the drug concentration used in Log molar scale. The calculated IC_50_ (5.62 nM) is given on the graph. (**h**) Bar graph demonstrating the proliferation of the OPCT-1 clones and parental OPCT-1 treated with control media, the docetaxel IC_50_ dose (5.5 nM) and double the docetaxel IC_50_ dose (11 nM) of parental OPCT-1, assessed using the thymidine proliferation assay. Data were analysed by means of a Factorial ANOVA using STATSTICA software *(p* = 0.014715*, F* < 2.318*, n* = 5).

Aldehyde dehydrogenase 1 (ALDH1), an enzyme involved in stem cell survival and early differentiation, has been used to identify both adult tissue stem cells and cancer stem cells, and several studies have associated ALDH1^hi^ populations with increased migration, drug resistance and tumourigenicity^37^,^38^,^39^,^40^,^41^,^42^. To further characterise our model, the ALDH1 activity of the clones and parental OPCT-1 was assessed using a commercially-available flow cytometry assay. Representative flow cytometric data of the clone with the smallest ALDH1^hi^ population, P5B3 (13.82%) and the clone with the largest ALDH1^hi^ population, P6D4 (45.45%), are shown in Fig. 6a. Interestingly, ALDH^hi^ populations were observed in each of the cell lines examined (Fig. 6b). Moreover, the differences in the percentage of ALDH1^hi^ cells among the clones were statistically significant (non-parametric, Kruskal-Wallis ANOVA, p < 0.0244, Kruskal-Wallis statistic <12.89). These flow cytometric data illustrate that ALDH1^hi^ cells presented as a shift in the population, rather than a distinct population of cells (Fig. 6a). This shift is typical of cells from solid malignancies and is consistent with the results observed with the recommended control cell line, SK-BR-3 (data not shown). The vimentin-low clone P5B3 exhibited the smallest population of ALDH1^hi^ cells (13.82%) and the vimentin-high clones P4B6 and P2B9 contained very high percentages of ALDH1^hi^ cells: 42.85% and 33.9%, respectively. Overall, this assay failed to demonstrate a direct correlation between EMT-associated marker-positivity and ALDH1 activity. Furthermore, the abundance of ALDH^hi^ cells observed brings the value of this assay in this model into question.

### Variable drug resistance of EMT derived clones

Drug resistance is a property which is attributed to cancer stem cells, and studies have also shown that EMT-generated mesenchymal cancer cells demonstrate resistance to chemotherapeutic agents^6^,^9^,^43^. We therefore investigated the chemotherapeutic sensitivity of OPCT-1 and the four clones. For this study, we selected docetaxel, the “standard of care” for patients with metastatic, castrate-resistant prostate cancer^44^. The half maximal inhibitory concentration (IC_50_) of parental OPCT-1 was determined using titrated concentrations of docetaxel and the ^3^H-thymidine proliferation assay. The docetaxel IC_50_ dose of parental OPCT-1 was calculated as 5.617 nM (Fig. 6g). We subsequently subjected the OPCT-1 clones and parental OPCT-1 to treatment with the determined IC_50_ dose, double the IC_50_ dose (11 nM) and medium alone, prior to assessing proliferation using the ^3^H-thymidine assay (Fig. 6h). Significant differences in the proliferation rates of the clones in response to different docetaxel treatments were observed (p < 0.014715, F < 2.318, Factorial ANOVA using STATISTICA software) (Fig. 6h). The mean inhibitory effect of each drug dose on proliferation was calculated as a percentage (Fig. 6h). This assay revealed that the vimentin-low clone P6D4 was the most resistant: only 10% inhibition with the IC_50_ dose and 14% inhibition with double the IC_50_ dose. Conversely, the vimentin-low clone P5B3 was the most sensitive to treatment with docetaxel: 41% inhibition with the IC_50_ dose and 60% inhibition with double the IC_50_ dose. Clones P6D4 and P4B6 were more resistant to docetaxel treatment than parental OPCT-1, whereas clones P5B3 and P2B9 were more sensitive than the parental cell line. These data demonstrate that cells capable of activating EMT do not necessarily exhibit enhanced resistance to treatment with chemotherapeutic agents.

### EMT-derived prostate cancer cells demonstrate enhanced migratory capacity, but are not necessarily more invasive

The *in vitro* scratch assay is a simple method for measuring cell migration^45^. The method usually involves creating a “scratch” in a cell monolayer, capturing images of the closure of the scratch and quantifying the migration rate of the cells. The assay showed that the clone P4B6 migrated significantly faster than all other and the four clones and the parental cell line (Fig. 6c and d), indicating a strong migratory phenotype. The cells that were stained with antibodies directed against vimentin and E-cadherin, demonstrated that vimentin-positive cells in the clones P2B9 and P4B6 migrated into the scratch (Fig. 6c–e). We did not observe any vimentin-positive cells in/along the scratches of clones P5B3, P6D4 and parental OPCT-1. Moreover, only clone P4B6 showed enhanced migratory capacity.

It is currently widely accepted that cancer cells induce the latent EMT programme in order to break away from the primary tumour, invade surrounding tissues and metastasise to distant sites^12^,^46^,^47^,^48^,^49^. As such, EMT is largely implicated in metastasis. We used our prostate cancer model to explore potential links between EMT and invasiveness. To that end, Matrigel invasion assays were conducted using the OPCT-1 clones and parental OPCT-1 in order to determine the percentage invasion for each (Fig. 6f). Our data revealed significant differences in invasiveness across the clones and parental OPCT-1 (non-parametric Friedman ANOVA, p < 0.0024, Friedman statistic <18.49). As anticipated, the most vimentin-positive clone (P4B6) was clearly the most invasive, with a median percentage invasion of 17.5%. (Fig. 6f). However, the second most vimentin-positive clone (P2B9) was the least invasive clone, with a median of 4.5% invasion. These data therefore indicate that cells that are capable of activating EMT are not inevitably more invasive than epithelial cancer cells.

### Cells capable of undergoing EMT are not necessarily more tumourigenic

**Figure 7:**
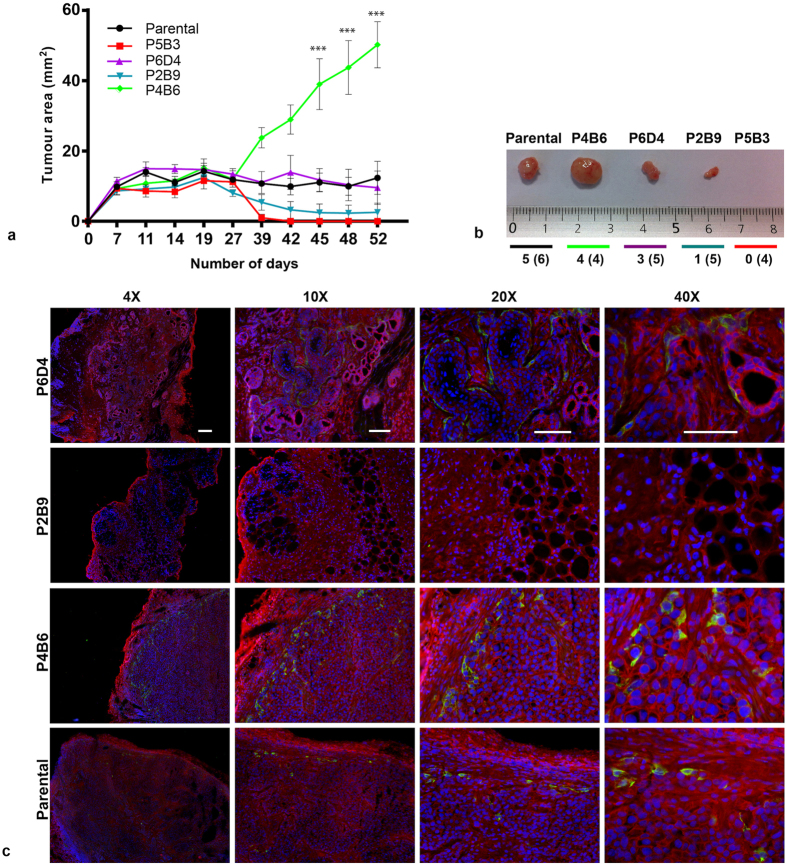
(**a**) *In vivo* tumourigenesis assay. Clones P5B3, P6D4, P2B9, P4B6 and parental OPCT-1 were injected subcutaneously into the right flanks of male athymic nude mice (6 animals per cell line). Tumour growth was monitored using calliper measurements and mice were euthanised once one of the tumours reached 1 cm in diameter. Data are presented as the mean tumour area ± SD. Statistical significance was calculated using the Student’s t-test (significance indicated as asterisks). (**b**) Representative tumours excised from tumour-bearing mice arranged in descending order of the clones’ *in vitro* vimentin positivity. (**c**) Immunofluorescent staining of tumour sections derived from clones P5B3, P6D4, P2B9, P4B6 and parental OPCT-1 for E-cadherin (red) and vimentin (green) expression. Representative images. (×10, ×20 and ×40 magnification, *n* = 3). Scale bar: 50 μM.

Cancer stem cells are believed to be solely responsible for tumourigenesis, tumour differentiation, tumour maintenance and tumour progression^50^. Several groups have identified, isolated and injected CSCs into immunocompromised mice in order to assess their tumourigenicity and differentiation potential compared with CSC-depleted populations^51^,^52^. In 2008, Mani *et al*., demonstrated that induction of EMT in immortalised human mammary epithelial cells generated cells with properties of stem cells^13^. We used the OPCT-1 clones to investigate a possible correlation between EMT and enhanced tumourigenesis in prostate cancer. After injecting 2.5 × 10^6^ cells per clone and parental OPCT-1 subcutaneously, we monitored tumour development over a period of 52 days (Fig. 7a). On completion of the experiment, the mice were euthanised, and the tumours were excised, snap-frozen in OCT, cryostat-sectioned and stained by immunofluorescence (Fig. 7c). Clear differences in the tumourigenicities of the clones were observed (Fig. 7a and b). Vimentin-high clone P4B6 formed tumours which were significantly larger (P ≤ 0.01) than those formed by the other clones and parental OPCT-1, in every mouse. Furthermore, this was the only clone to form tumours in all of the mice injected (Fig. 7b). In contrast, clone P2B9 only formed a tumour in one of five mice. In keeping with the *in vitro* data, which revealed a non-aggressive phenotype, clone P5B3 failed to form tumours in any of the six mice injected. Interestingly, clone P6D4 was the second most tumourigenic of the clones and formed tumours in three out of five mice. Sections were stained using an antibody to murine MHC class I H2^Kd^ mouse MHC molecule, which confirmed their human origin (Supplementary Figure 8).

Immunofluorescence staining of the sectioned tumours revealed patterns of E-cadherin and vimentin protein expression which were not consistent with what had been observed in *in vitro* cultured cells (Fig. 7c). Intriguingly, clone P6D4 formed gland-like structures with areas of high E-cadherin positivity, as well as structures surrounded by vimentin-positive cells. Moreover, vimentin-positive cells were not observed in the tumour formed by clone P2B9. It is important to note that the “vacuoles” observed in the P2B9 tumour are likely to have been artefacts created during sectioning, as this tumour was the smallest and most difficult to section. Clone P4B6 and parental OPCT-1 formed tumours with tight cell-cell contact and fewer glandular structures than clone P6D4. These cells formed tumours with vimentin-positive cells predominantly located along the periphery.

These data revealed that the two clones capable of activating EMT (P4B6 and P2B9) behaved very differently *in vivo*. Despite the fact that the most vimentin-positive clone (P4B6) was more tumourigenic than the parental cell line, taken together this experiment failed to demonstrate a direct correlation between EMT and enhanced tumourigenesis in prostate cancer.

## Discussion

Cancer cell plasticity and cancer stem cells remain largely elusive topics in cancer biology. Although EMT has been studied extensively using breast cancer cell lines and murine models, there is a need to develop our understanding of the role and properties of EMT in other human malignancies, including prostate cancer. Herein, we report the derivation and interrogation of phenotypically distinct clones from a primary prostate cancer cell line with the ability to activate the EMT programme, without artificial induction.

One of the main limitations of prostate cancer research is the lack of availability of cell lines that are derived from primary prostate carcinoma^53^. The commonly used cell lines PC3 and DU145 are derived from metastatic tumour lesions of bone and brain, respectively. Our data reveal that these cell lines are not appropriate for the study of spontaneous EMT events in prostate cancer, and we identify the primary cell-derived OPCT-1 cell line as a suitable alternative.

The induction of EMT in cultured cancer cells has been achieved using a variety of methods, including ectopic expression of transcription factors^13^,^54^, growth factors^35^, and enzymes^55^, incubation with growth factors^56^, cytokines^57^, enzymes^58^ and androgen deprivation^16^. Indeed, to date, the vast majority of EMT studies in cancer have examined artificially-induced EMT-derived cells. To our knowledge, we are the first to derive and interrogate a spontaneous model of prostate cancer EMT.

We derived four phenotypically distinct clones from the OPCT-1 cell line and investigated a number of features which have been attributed to cancer stem cells. Examination of several EMT-associated markers at both the mRNA and protein level revealed that only two of the clones were capable of activating the EMT programme. Based on previous findings, we anticipated that the two EMT-positive clones (P4B6 and P2B9) would exhibit an aggressive/stem cell phenotype, as characterised by enhanced sphere-forming ability, heightened resistance to chemotherapeutic agents, expression of stem cell markers, high ALDH activity, enhanced clonogenicity, and tumour-forming capacity *in vivo*^1^,^39^,^59^. We were intrigued to discover that P4B6 exhibited an aggressive and stem cell-like phenotype, whereas clone P2B9 was non-aggressive, and even less aggressive/stem cell-like than the EMT-negative clone P6D4. Due to this disparity, we were unable to confirm that EMT of prostate cancer cells generates cells with the properties of stem cells, as it was evident that this statement could not always be substantiated.

Based on the biological context in which they occur, EMTs have been classified into three subtypes^48^. Type 1 EMT events are critical for implantation, embryogenesis and organ development, Type 2 EMT events are activated in the context of inflammation; they are associated with wound healing, tissue regeneration and organ fibrosis, and Type 3 EMT events are involved in tumour progression and metastasis. Unlike Type 1 and Type 3 EMT events, which generate mesenchymal cells, Type 2 EMT events generate fibroblasts from mature epithelial cells^59^,^60^. We have shown that EMT of prostate cancer cells can give rise to distinct progeny, with contrasting characteristics. The highly aggressive stem-like phenotype displayed by clone P4B6 is consistent with several studies which have implicated EMT in the generation of CSCs^13^,^14^,^35^. In contrast, the non-aggressive characteristics of clone P2B9 demonstrate that EMT of prostate cancer cells does not necessarily generate cells with the properties of stem cells. Our data therefore reveal that Type 3 EMT events can be further categorised into events which give rise to aggressive stem-like cells and those which generate non-aggressive, non-stem-like progeny.

In 2005, Collins *et al*., identified putative prostate cancer stem cells using a panel of markers which had been previously exploited to identify normal prostate stem cells (CD44, CD133, Integrin α2β1)^27^. Although the EMT-positive clone P4B6 clearly demonstrated stem cell properties (as indicated by high expression of CD44, Nanog, Oct4 and Sox2 in addition to their high performance in *in vitro* and *in vivo* assays), we observed that the EMT-derived, vimentin-positive cells did not express Integrin α2β1.

High expression of CD44 with low/no expression of CD24 in prostate cancer cells has been reported to have high tumour initiating capacity in prostate cancer cell models^28^. From the surface expression studies of these molecules in the OPCT-1 parental and clonal progenies, we observed that the reported cancer stem cell population was present in the parental cell line but not in any other clones, irrespective of their high tumourigenic potential (particularly lacking in clone P6D4 and P4B6). Also, CD44 and CD24 surface expression levels varied significantly, with high levels of CD44 expression evident in both clones comprising mesenchymal populations (P2B9 and P4B6). High surface expression of CD24 was observed in OPCT-1 (Parental), clone P6D4 and P4B6, which were also highly tumourigenic in the mouse xenograft model. As such, this study supports the concept that there are multiple stem-like aggressors in cancer, and thereby emphasises the importance of a multiple-approach strategy to combatting cancer, rather than focussing on one population (such as CSCs).

CD44 isoform class switching (CD44v to CD44s) has been reported in prostate and breast cancer cell lines as a mechanism of EMT^61^,^62^. As with the FACS data, Western blotting also showed an overall decrease in CD44 expression levels in the parental, P5B3 and P6D4 cell lines, with a weak band at 80 kDa indicating the CD44s isoform. However, CD44s expression was found to be increased in the two clones, P2B9 and P4B6. This high expression of the CD44s isoform in the clones comprising EMT-derived cells was in agreement with existing literature that CD44s expression increases with mesenchymal characteristics^62^. However, we have also noted the presence of the CD44v isoform (associated with the epithelial phenotype) in the same clones, which is consistent with the fact that these clones comprise a mixed population of epithelial and mesenchymal cells.

While the argument of increased epithelial traits holds true in terms of CD44v expression, the clones comprised of epithelial and mesenchymal populations were fundamentally different in their membrane expressed E-cadherin (an indicator of functional epithelial cells). Considering the lack of membrane inserted E-cadherin (a critical component of cell-cell adhesion) and with noticeable EMT events, the low invasive and migratory properties observed with clone P2B9 in the wound healing and trans-well assays were surprising. In contrast to clone P2B9, clone P4B6 demonstrated the highest levels of membrane inserted E-cadherin (an indicator of functional epithelial characteristics) in their epithelial compartment (Supplementary Figure 3). This clone is also highly tumourigenic and shows high expression of stem cell/pluripotent genes (Fig. 4) along with other functional stem cell traits (Fig. 5). Clone P4B6 possesses stem-cell characteristics in addition to non-exogenously induced EMT capacity in 2D and sphere-forming culture conditions. These features may account for its aggressive/high tumourigenic potential consistent with the observations made by Mani *et al*., (2008)^13^, whereas the second most tumourigenic clone P6D4, did not have any observed EMT events in 2D culture systems. However, P6D4 stained positive for vimentin only in the tumours (Fig. 7), indicating that these cells might be more prone to undergoing EMT in the presence of exogenous stimuli.

Contrasting evidence about the relationship between EMT and cancer stem cells exists in the literature. While several studies using induced models of EMT have demonstrated a positive correlation between EMT and cancer stem cell traits^13^, other studies have convincingly demonstrated in prostate and bladder cancer that the loss of epithelial characteristics leads to a decrease in self-renewal and pluripotency characteristics and reduced metastatic potential^63^. In this study, the most tumourigenic clone P4B6 demonstrated tumour-initiating and invasive properties, thereby indicating that this clone may be epigenetically programmed for undergoing EMT while retaining its core stem cell characteristics. Moreover, the co-operation between the epithelial and transitioned mesenchymal components within this clone may be another contributing factor for its aggressive characteristics. It is also worth noting that the absence of the expression of a key marker of prostate cancer stem cells, Integrin α2β1, in vimentin-positive cells in clone P4B6, may support the observation made by Celià-Terrassa *et al*., that EMT can suppress major attributes of epithelial tumour-initiating cells^63^.

While EMT has been widely accepted in the dissemination of cancer cells from the primary site, the repopulation of these cells in distant organs would require tumour initiating capacity in a completely different environment. Two independent studies by Tsai *et al*. and Ocana *et al*. in 2012^64^,^65^, with two elegant mouse models of metastasis, showed the importance of EMT in cancer cell dissemination from the primary tumour (extensive review in Nieto *et al*.^66^). However, successful recolonisation of circulating tumour cells in the lung required the acquisition of epithelial characteristics or otherwise MET (mesenchymal to epithelial transition). Suppression of epithelial characteristics in mesenchymal subpopulations has been shown to lead to the loss of tumour-initiating characteristics^63^,^64^ indicating the decoupling of stem cell properties from EMT characteristics when cells acquire mesenchymal status. We have shown that cells capable of activating the EMT programme can lack stem cell and invasive traits (clone P2B9), indicating the existence of two key parameters for a metastatic phenotype in that clone. This suggests that the EMT occurring in P4B6 might be transient and thereby preserving the self-renewal potential, as proposed by Celia-Terrassa *et al*.^63^. Cell cooperativity proposed by Tsuji *et al*. which may have relevance to our model, introduced that co-operation between EMT and non-EMT cells is necessary for an effective metastatic process^67^. Their model proposed that mesenchymal cells help to break the surrounding tissue, which enables the non-EMT cells to escape into the blood stream and only the epithelial cells, which retain self-renewal properties, can establish well differentiated metastases.

Though the complete biological mechanisms governing the spontaneous EMT events observed in this model have not been fully elucidated, the available data suggests that there may be a link between the expression of transcription factor ZEB1 and CD44s in the most aggressive clone, P4B6. ZEB1 and CD44s expression was found to be lower in P2B9 compared to P4B6. We anticipate ZEB1 expression still plays a significant role in EMT in both clones, however, the quantitative levels (relatively higher in clone P4B6 compared to P2B9) determine the aggressive phenotype^68^. The complete picture of the molecular mechanisms governing the phenotypic differences observed between the clones will only be elucidated though complete transcriptomic/proteomic profiling of these cells.

The model developed in the present work provides a valuable resource for the investigation of EMT in human prostate cancer. Unlike artificially-induced models of EMT, the EMT-derived cells in this study express endogenous levels of EMT-associated proteins. As such, these clones can be utilised to derive an authentic EMT-signature, which could be extended to clinical applications. Furthermore, the OPCT-1 clones have been characterised both *in vitro* and *in vivo;* thus, can be utilised to identify the molecular signatures that confer aggression. These clones provide a significant resource and considerable scope for future studies that are focussed on interrogating the fundamental mechanisms involved in cancer progression. They will also be of significant value for studies based on biomarker discovery and the identification of ‘druggable’ or immunotherapeutic targets that can be exploited for the development of new approaches for the treatment and management of prostate cancer.

## Methods

The overall workflow for this study is given in Fig. 1.

### Cell Culture

The primary disease-derived prostate cancer cell lines P4E6 (kindly provided by Professor N. Maitland, The University of York) and OPCT-1 and OPCT-2 (generously donated by Onyvax Ltd) were maintained in KSFM media (Gibco) supplemented with 2% (v/v) FCS. P4E6 were maintained in the same media, also containing pituitary extract (50 μg/mL) and epidermal growth factor additives (5 ng/mL). The description of P4E6 has been published elsewhere^69^. Derivation of OPCT-1 and OPCT-2 has been previously briefly described^70^,^71^ and the details of these cell lines are also available from Asterand Bioscience. OPCT-1 has been used previously in the literature^72^,^73^,^74^. Briefly, OPCT-1 cell lines are derived from a 68 year old patient (TNM Stage T1cN0M0; Gleason score of 6 [3 + 3]) and OPCT-2 were derived from a 58 year old patient (T2aN0M0 and Gleason score of 5 [2 + 3]) and immortalised via replication-defective retrovirus transferring the transforming HPV16 or HPV18 E6 and E7 genes. Both the cell lines were characterised as tumour derived with its chromosomal instability and various other assays and we have independently confirmed the reported chromosomal abnormalities in our OPCT-1 cell line by karyotyping. All cells were incubated in a 5% (v/v) CO_2_-humidified atmosphere at 37 °C. Cells were harvested using Trypsin-Versine (Lonza). The metastatic disease-derived human prostate cancer cell lines, PC3 and DU145, were purchased from the American Type Culture Collection (ATTC). PC3 cells were maintained in HAM’S F12 media (Lonza) supplemented with 10% (v/v) foetal calf serum (FCS), 1% (w/v) L-glutamine (Lonza) and 1% (v/v) non-essential amino acids (NEAA) (Lonza). DU145 cells were maintained in DMEM media (Lonza) supplemented with 10% (v/v) FCS, 1% (w/v) L-glutamine, 1% (v/v) NEAA and 1% (v/v) sodium pyruvate (Lonza).

### Immunofluorescence

Cells were washed and fixed with 4% (w/v) paraformaldehyde prior to incubation with primary monoclonal and polyclonal antibodies: murine anti-E-cadherin DH01 monoclonal antibody (mAb) (clone DCS-266, Invitrogen), murine anti-cytokeratin pan polyclonal antibody (Sigma), rabbit anti-vimentin mAb (clone SP20, Abcam), murine anti-fibronectin mAb (clone IST-9, Abcam), murine anti-N-Cadherin mAb (clone 32/N-Cadherin, BD Biosciences), goat polyclonal anti-SNAI1 (E-18) antibody (Santa Cruz Biotechnology), goat polyclonal anti-SLUG (D-19) antibody (Santa Cruz Biotechnology), mouse IgG isotype control (AbD Serotec), rabbit IgG isotype control (Pierce Biotechnology), goat IgG isotype control (Pierce Biotechnology) diluted in blocking solution (1X DPBS-0.1% (v/v) TWEEN containing 10% (w/v) BSA) for one hour at room temperature. Cells were subsequently washed and incubated with the secondary antibodies (chicken anti-rabbit Alexa Fluor 488, donkey anti-goat Alexa Fluor 568, goat anti-rabbit Alexa Fluor 488, goat anti-mouse Alexa Fluor 568 (Thermo Scientific), diluted in blocking solution, for a further one hour at room temperature. After washing, the coverslips were mounted in VECTASHIELD^®^ mounting medium with DAPI (Vector Laboratories) and imaged using an Olympus BX51 fluorescence microscope. Following examination of the five prostate cancer cell lines using the aforementioned panel of antibodies against well-established epithelial and mesenchymal markers, OPCT-1 was selected for further study. All proceeding methods were conducted on the OPCT-1 cell line only.

### Derivation of OPCT-1 clones

Single-cell clones were derived from the OPCT-1 cell line by introducing the cells into 96 well plates at a dilution yielding <1 cell per well (0.33 cells per well). The wells were microscopically examined to ensure that only one cell had been seeded per well. Plates were maintained at 37 °C in a 5% (v/v) CO_2_-humidified atmosphere and were examined twice a week for the presence of single colonies. After 21 days, 51 clones were transferred from the 96 well plates into six well plates and these were subsequently screened for the expression of CD44, E-cadherin and vimentin by immunofluorescence. Four clones with different E-cadherin and vimentin expression profiles were selected for further study, and these were subsequently re-screened for the expression of E-cadherin and vimentin after further passaging. The phenotypic stability of the four OPCT-1 clones, on the basis of E-cadherin and vimentin expression, was monitored by immunofluorescence throughout the study.

### Assessment of membrane localised E-cadherin

Stacked confocal images were generated for all the clones and the parental cells using anti E-cadherin antibody (Cell Signalling Technologies, Rabbit, Clone 24E10). All the clones were fixed and permeabilised prior to the staining as previously described. The images were acquired using confocal microscope (Leica TCS SP5 confocal microscope). FITC conjugated anti-rabbit IgG secondary antibody (DAKO) was used for the staining detection. A total of three images were acquired from three independent wells with HP PL FLUOTAR 20.0 × 0.50 dry objective to cover enough field of view with sufficient number of cells (0.84 μM step size with 30–40 sliced images stacked). Cells with membrane expression of E-cadherin was scored using three independent images from each clones (300 cells).

### Cell Sorting – Re-cloning of OPCT-1 clones

Single-cell suspensions of the four OPCT-1 clones were sorted into fluorescence-compatible 96 well plates (BD Biosciences) at a density of one cell per well (32 wells per clone), using a Beckman Coulter MoFlo XDP High-Speed Cell Sorter. The plates were incubated at 37 °C, 5% (v/v) CO_2_ for 14 days, after which the cells were fixed with 4% (w/v) paraformaldehyde, stained for the expression of E-cadherin and vimentin and imaged using an Olympus BX51 fluorescence microscope. This assay was conducted on three separate and independent occasions.

### Flow Cytometry

Cells were harvested by trypsinisation, counted using the trypan blue exclusion method, and 1 × 10^6^ cells were transferred to 12 × 75 mm polycarbonate tubes. The cells were then washed twice in 2 mL flow cytometry buffer (0.5% (w/v) BSA in DPBS) by centrifugation at 400 g for three min at 4 °C. Cells were then fixed in 2% (w/v) paraformaldehyde for 10 min at 37 °C, washed, and then permeabilised with 1 mL 90% (v/v) ice-cold methanol on ice for 30 min. Following permeabilisation, the cells were washed and then incubated with a PE-conjugated murine anti-vimentin mAb (clone RV202, BD Biosciences) or the corresponding PE-conjugated mouse isotype control (IgG1κ-PE, BD Biosciences), in accordance with the manufacturer’s instructions. CD44 and CD24 staining was achieved by staining the cells without permeabilisation using FITC conjugated antiCD44 (eBioscience, clone 24E10) and APC conjugated CD24 (eBioscience, clone eBioSN3) antibodies. Prior to flow cytometric analysis, the cells were washed twice and then re-suspended in 300–400 μL of Isoton solution (Beckman Coulter) on ice. Cells were analysed using a Beckman Coulter Gallios flow cytometer. Histograms and dot plots were derived and analysed using Beckman Coulter Kaluza version 1 software.

### Western Blot Analysis

Total cell lysates were prepared for each of the four OPCT-1 clones and parental OPCT-1 by lysing the cells in the presence of 50 mM Tris, pH 7.5, 5 mM EDTA and 1% (w/v) SDS on ice. Next, 30 μg of total protein from each sample was prepared with sample reducing buffer (0.5 M Tris-HCL (pH 6.8), 2% (w/v) SDS, 10% (v/v) glycerol, 1% (w/v) DTT) at a ratio of 3:1 (lysate: reducing buffer) and was resolved on an SDS gel with Tris/Glycine/SDS gel running buffer (Geneflow). The samples were subsequently transferred onto nitrocellulose membranes prior to probing with mouse anti-E-cadherin DH01 mAb (clone DCS-266, Invitrogen), rabbit anti-vimentin mAb (clone SP20, Abcam), murine anti-fibronectin mAb (clone IST-9, Abcam), murine anti-N-Cadherin mAb (clone 32/N-Cadherin, BD Biosciences), murine anti-CD44 mAb (clone 156-3C11, Cell Signalling Technology) and murine anti-β-actin mAb (clone AC-74, Sigma). After probing with the primary antibodies, the membranes were washed and probed with horseradish peroxidase (HRP) conjugated goat anti-mouse or rabbit anti-goat polyclonal antibodies (Dako), as appropriate. The membranes were then washed and exposed using RapidStep ECL reagent (Calbiochem) for times that depended on the abundance of the protein (typically ~1 minute), before being imaged using a CCD camera (Fuji Systems).

## RNA Isolation, cDNA Synthesis and qRT-PCR

RNA isolation from cultured cells was executed using RNA Stat-60 (Amsbio, UK). Cells were grown to 80% confluence and the medium was completely removed before cell lysis. Isolations were performed according to manufacturer protocols, without any modifications. Harvesting of cells using trypsin/EDTA prior to RNA isolation was avoided in order to minimise potential effects on antigen expression. The RNA pellets were dissolved in 30 μL of nuclease-free water (Ambion), and purified using the RNeasy Mini kit (Qiagen). RNA quantity and quality was assessed using a NanoDrop 8000, and further confirmed using an Agilant Bioanalyzer and RNA6000 nano kits. cDNA synthesis was performed using Superscript III (Invitrogen-Life Technologies) reverse transcriptase, according to the manufacturer guidelines; using 2 μg of total RNA and 0.2 μg of oligo (dT)_15_ (Promega). All primer sequences were either selected from previously published literature, or, if unavailable in the published literature, designed using Primer 3.0 software (v. 0.4.0, Whitehead Institute for Biomedical Research, Massachusetts, USA). Primers were synthesised from Eurofins MWG Operon (Germany) at HPSF purity. Pre-designed primers for basal, intermediate and luminal characterisation of the cell lines were purchased from Sigma Aldrich (KiCqStart SYBR Green Primers). The primers used for the study are provided in Supplementary Table 2. Real-time quantitative PCR was employed to examine the gene expression patterns using SYBR Green Master Mix (Bio-Rad), according to the manufacturer’s instructions. Thermal cycling was performed using a Rotor-Gene PCR cycler (Qiagen). The efficiencies were calculated using Rotor-Gene software.

### Sphere-Forming Assay

Preliminary studies optimised this assay in terms of medium composition, plate format, cell density and duration of culture. Single-cell suspensions were plated in triplicate wells of an ultra-low adherent 24-well plate at a density of 10,000 cells per well, and were cultured in KSFM 2% (v/v) FCS at 37 °C, 5% (v/v) CO_2_. After 12 days, spheres were observed microscopically and counted by two individuals; an average of the counts was taken. This assay was conducted in triplicate wells in three separate experiments.

### Clonogenic Assay

Single-cell suspensions were plated at the clonal density of 125 cells per well of a six-well plate, two wells per clone. Following ten days in culture, the colonies were fixed for 15 min at 4 °C with 4% (w/v) paraformaldehyde and stained with crystal violet solution (0.5% (w/v) crystal violet in 70% (v/v) ethanol) for 15 min at room temperature. The colonies were washed with DPBS and allowed to dry, prior to counting under a light microscope. Colonies with fewer than 32 cells were excluded from the counts. This assay was conducted in duplicate wells in three separate experiments.

### Aldefluor Assay

The Aldefluor stem cell assay (Stem Cell Technologies) was conducted in accordance with the manufacturer’s instructions. In order to avoid loss of staining, as a result of ALDH1 efflux from the cells, samples were kept covered on ice at all times and analysed immediately using a Beckman Coulter Gallios flow cytometer. Furthermore, the order in which the cells were analysed was changed in each repeat of the experiment. This assay was conducted on four separate occasions. Histograms and dot plots were derived using the Beckman Coulter Kaluza version 1 software. The percentage of ALDH1^hi^ cells was determined by gating around a density plot of control cells (treated with DEAB reagent), applying the same gate to the test cells and subtracting the number of cells within the control gate from the number of cells within the test gate. The mean percentage of ALDH1^hi^ cells present in each clone, as compared with parental OPCT-1, was calculated.

### [^3^H]-Thymidine Incorporation Proliferation and Drug Assay

Briefly, 1 × 10^4^ OPCT-1 cells were plated in 32 wells of a 96 well plate and incubated for approximately 20 h at 37 °C, 5% (v/v) CO_2_. Following incubation, the supernatant was removed and replaced with 200 μL control medium (medium only) and medium containing docetaxel at 1, 3, 10, 30, 100, 300, 1000 nM. The plate was then incubated for 48 h at 37 °C, 5% (v/v) CO_2_. Following incubation, [^3^H]-Thymidine (final concentration of 0.037 MBq/mL) was added to the cells and the cells were incubated for a further 20 h at 37 °C with 5% (v/v) CO_2_. Culture supernatants were removed using a Filtermate Harvester (Perkin-Elmer), following which, cells were incubated for 15 min with 30 μL of Trypsin-Versine, prior to being harvested onto the Unifilter plate (Perkin-Elmer). The plate was allowed to dry for 2 h at room temperature before being overlaid with 40 μL of Microscint-O scintillation fluid and analysed using an NXT Top-Count microplate scintillation counter. This assay was conducted in quadruplicate on five separate occasions. The data obtained were used to plot a dose-response curve and ascertain the docetaxel IC_50_ for parental OPCT-1, using GraphPad Prism 5 software.

The docetaxel IC_50_ for parental OPCT-1 was determined as 5.617 nM. To determine their relative sensitivities to the drug, the proliferation of the OPCT-1 derived clones was assessed in normal media and media containing 5.5 nM and 11 nM docetaxel, using the approach described above.

## Invasion and Migration Assays

### Scratch Migration Assay

The invasive assays were performed using a classic wound healing assay. The cells were seeded and grown to confluency in a 24 well tissue culture plate. Cells were treated with 10 μg/mL of mitomycin C for 2 hours (concentration determined previously as optimal for cell growth arrest and viability. Supplementary Figure 6). Cells were washed three times with sterile PBS and replaced with normal tissue culture media with 2% serum prior to wounding. The wound was imaged at 0 and 24 h at the same field of vision with the help of a guide-line previously drawn underneath the plates (Carl Zeiss, 5X magnification). The wound area was calculated using Axiovision REL software (version 4.8.1.0) and the % of wound closure was calculated from time 0 to 24 h. For immunofluorescence staining of the migratory cells, fluorescence-compatible 96-well plates (BD Biosciences) were coated by incubation with 30 μL poly-L-lysine (Sigma Aldrich) per well for one hour. Unbound poly-L-lysine was removed from each well prior to seeding cells in quadruplicate wells at the high density of 5 × 10^4^ cells per well. After 24 h, 200 μL pipette tips coated with poly-L-lysine were used to create a scratch in the confluent monolayer, at the centre of each well. The cells were washed twice with sterile DPBS, prior to being cultured in serum-free KSFM media for 12 h at 37 °C, 5% (v/v) CO_2_. Cells were then fixed with 4% (w/v) paraformaldehyde for 15 min at 4 °C, and their expression of E-cadherin and vimentin was determined by immunofluorescence, as described previously. This assay was performed on three separate occasions.

### *In Vitro* Matrigel Invasion Assay

This assay was conducted using 24 well Biocoat Matrigel Invasion Chambers containing BD Falcon Cell Culture Inserts with 8 μm pore-size PET membranes that had been treated with Matrigel matrix in accordance with the manufacturer’s instructions (BD Biosciences), with an additional step introduced to optimise the counting procedure. Cells were stained with propidium iodide (PI) (0.1 mg/mL) prior to counting the invading cells. Plates were then scanned and imaged using an ELISPOT plate reader (Cellular Technology Ltd.), and the images were analysed using ImmunoSpot software (n = 3).

## ***In vivo*** tumourigenesis

Thirty male athymic nude mice (six weeks of age), were purchased from Harlan Laboratories, UK. Animals were housed and experiments performed in accordance with Animals (Scientific Procedures) Act 1986 (UK Home Office regulations), under Nottingham Trent University Project License (PPL) 40/3563 and the study protocols approved by the institutional Project License holder, in a pathogen-free animal facility. Cells were injected at a density of 2.5 × 10^6^ with 100 μL 1:6 serum-free KSFM medium: Matrigel (BD Biosciences), subcutaneously into the right flank (6 mice per cell line/clone). Tumour development was measured twice weekly using calliper measurements until one of the tumours reached a diameter of 1 cm, at which time the experiment was terminated. Tumours were then extracted, mounted on cork boards with OCT, snap-frozen in liquid nitrogen-cooled 2-butanol and stored at −80 °C, prior to cryostat sectioning.

After sectioning the tumours onto sialinised slides at a thickness of 6 μm, the specimens were fixed with 4% (w/v) paraformaldehyde for 15 min at 4 °C, and the expression of E-cadherin and vimentin was determined by immunofluorescence, as previously described. In order to rule out the murine stromal contamination in the implanted tumours, all the sections were stained with murine MHC class I H-2Kd antibody (BioLegend SF1-1.1).

## Statistical analysis

Unless stated, data are presented as mean ± standard error of the mean (SEM), or median ± interquartile range, where percentages are presented. Data are presented as overall significance between the clones and parental OPCT-1 in a given test represented, in some figures, by asterisks with the significance levels p ≤ 0.05 (*), p ≤ 0.01 (**), and p ≤ 0.001 (***). When investigating overall significance between the clones and parental OPCT-1, non-parametric testing was performed using the Friedman test, for instances in which the same number of replicates was used, or a Kruskal-Wallis test, when the number of replicates varied. Significant differences between the clones and parental OPCT-1 were investigated using the Dunn’s Multiple Comparison (Post-Hoc) test.

## Additional Information

**How to cite this article**: Harner-Foreman, N. *et al*. A novel spontaneous model of epithelial-mesenchymal transition (EMT) using a primary prostate cancer derived cell line demonstrating distinct stem-like characteristics. *Sci. Rep.* **7**, 40633; doi: 10.1038/srep40633 (2017).

**Publisher's note:** Springer Nature remains neutral with regard to jurisdictional claims in published maps and institutional affiliations.

## Supplementary Material

Supplementary Information
