# Supplementary material for: A novel spontaneous model of epithelial-mesenchymal transition (EMT) using a primary prostate cancer derived cell line demonstrating distinct stem-like characteristics
Source: Sci Rep. 2017 Jan 17;7:40633. doi: 10.1038/srep40633 (PMC5240554; doi:10.1038/srep40633)
Supplement: Supplementary Information [file srep40633-s1.pdf]

Supplementary Information:

A novel spontaneous model of epithelial-mesenchymal transition (EMT) using a primary prostate cancer derived cell line demonstrating distinct stem-like characteristics

Naomi Dunning-Foreman, Jayakumar Vadakekolathu, Stéphanie A. Laversin, Morgan G. Mathieu, Stephen Reeder, A. Graham Pockley, Robert C. Rees, David J. Boocock

Supplementary Figure 1

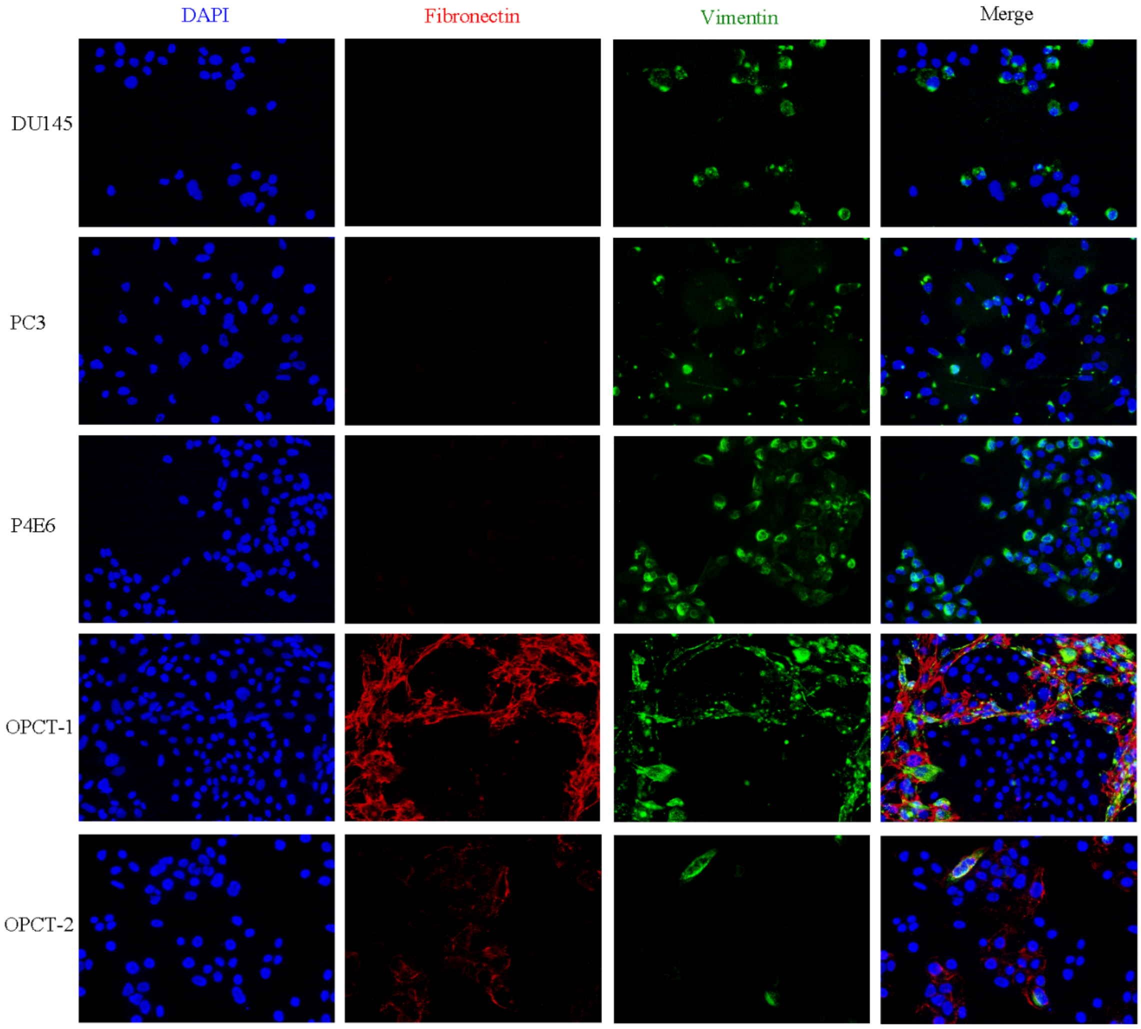

Dual immunofluorescent staining of DU145, PC3, P4E6, OPCT-1 and OPCT- 2 using a murine monoclonal antibody against cellular fibronectin and a rabbit polyclonal antibody against the mesenchymal marker, vimentin. E-cadherin was labelled with an anti-mouse Alexa Fluor®568-conjugated secondary antibody and vimentin was labelled with an anti-rabbit Alexa Fluor®488-conjugated secondary antibody. Nuclear staining was achieved using mounting media with DAPI. Representative images. Image magnification at x20.

Supplementary Figure 2:

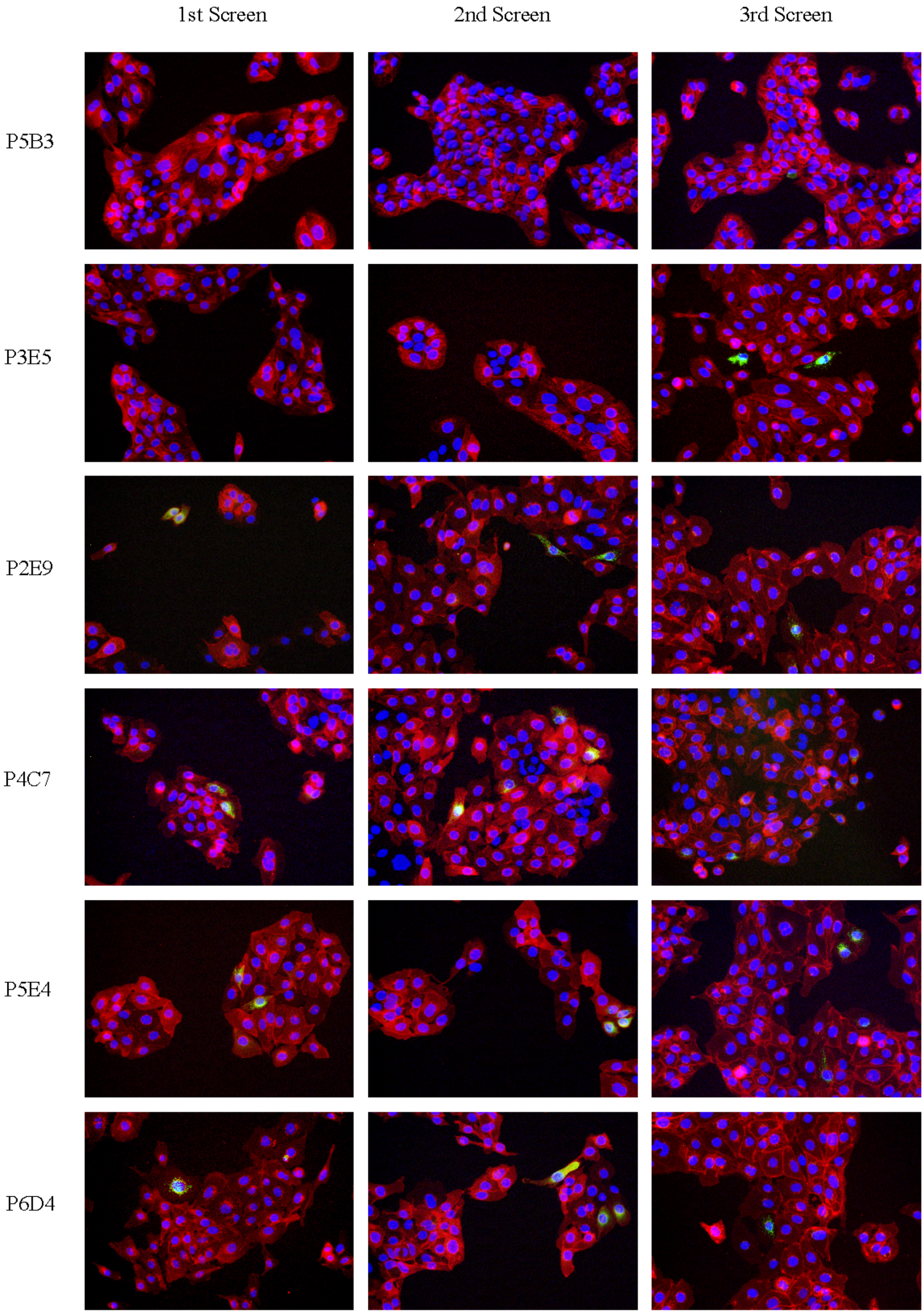

Supplementary Figure 2 (Continued)

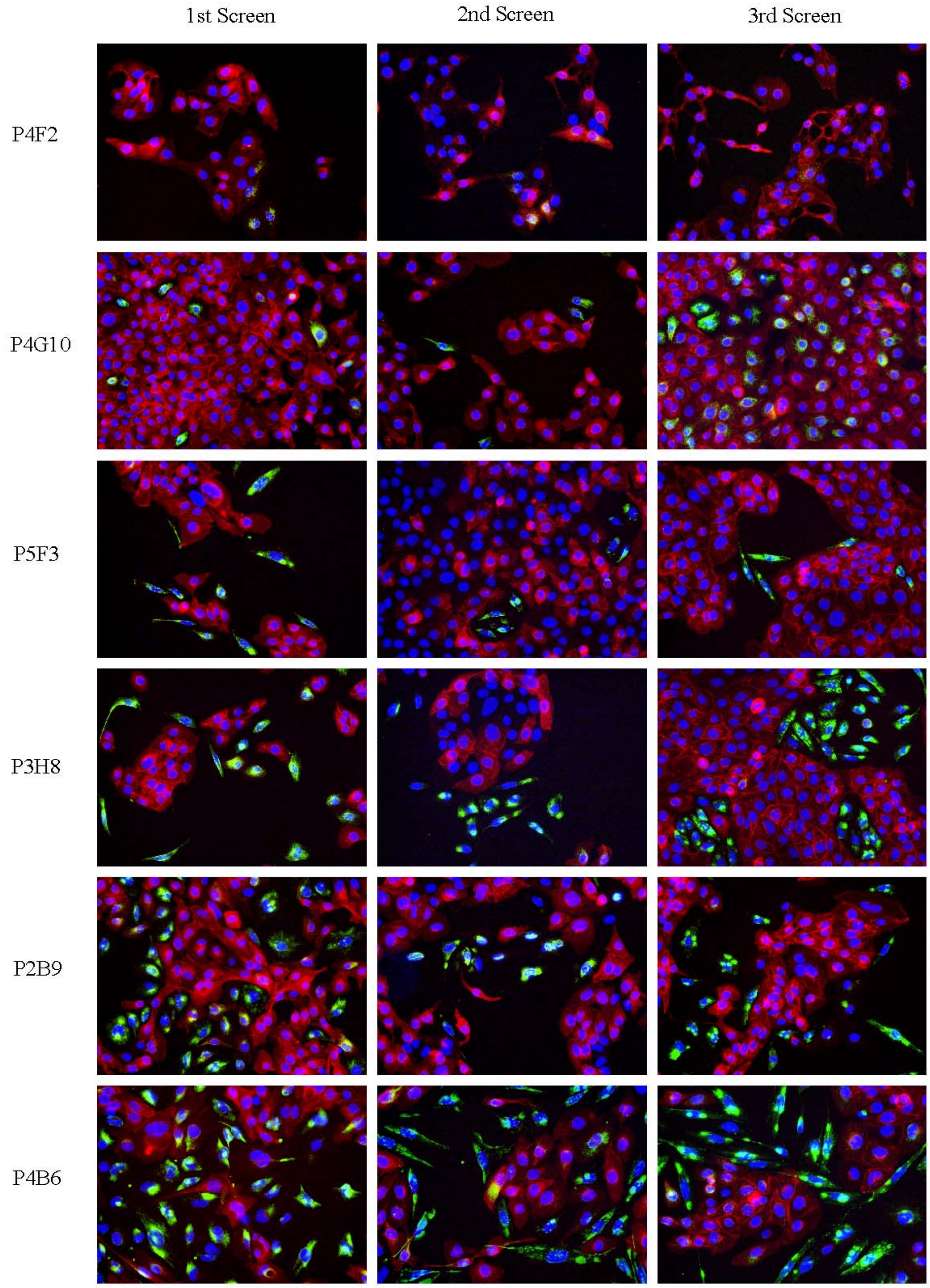

Supplementary Figure 2: *Dual immunofluorescent staining of 1-12 OPCT-1 clones of interest; P5B3, P3E5, P2E9, P4C7, P5E4, P6D4, P4F2, P4G10, P5F3, P3H8, P2B9 and P4B6, using a murine monoclonal antibody against the epithelial marker, E-cadherin and a rabbit polyclonal antibody against the mesenchymal marker, vimentin. E-cadherin was labelled with an anti-mouse Alexa Fluor®568-conjugated secondary antibody and vimentin was labelled with an anti-rabbit Alexa Fluor®488-conjugated secondary antibody. Nuclear staining was achieved using mounting media with DAPI. 1st screen represents the initial screening of the 51 clones, from which these clones were selected (P2), 2nd screen represents the second screening conducted after defrosting and passaging the cells (P3) and 3rd screen represents the third screening conducted after defrosting and passaging the clones for a second time (P4). (n=3). Representative images. Image magnification at x20.*

Supplementary Figure 3

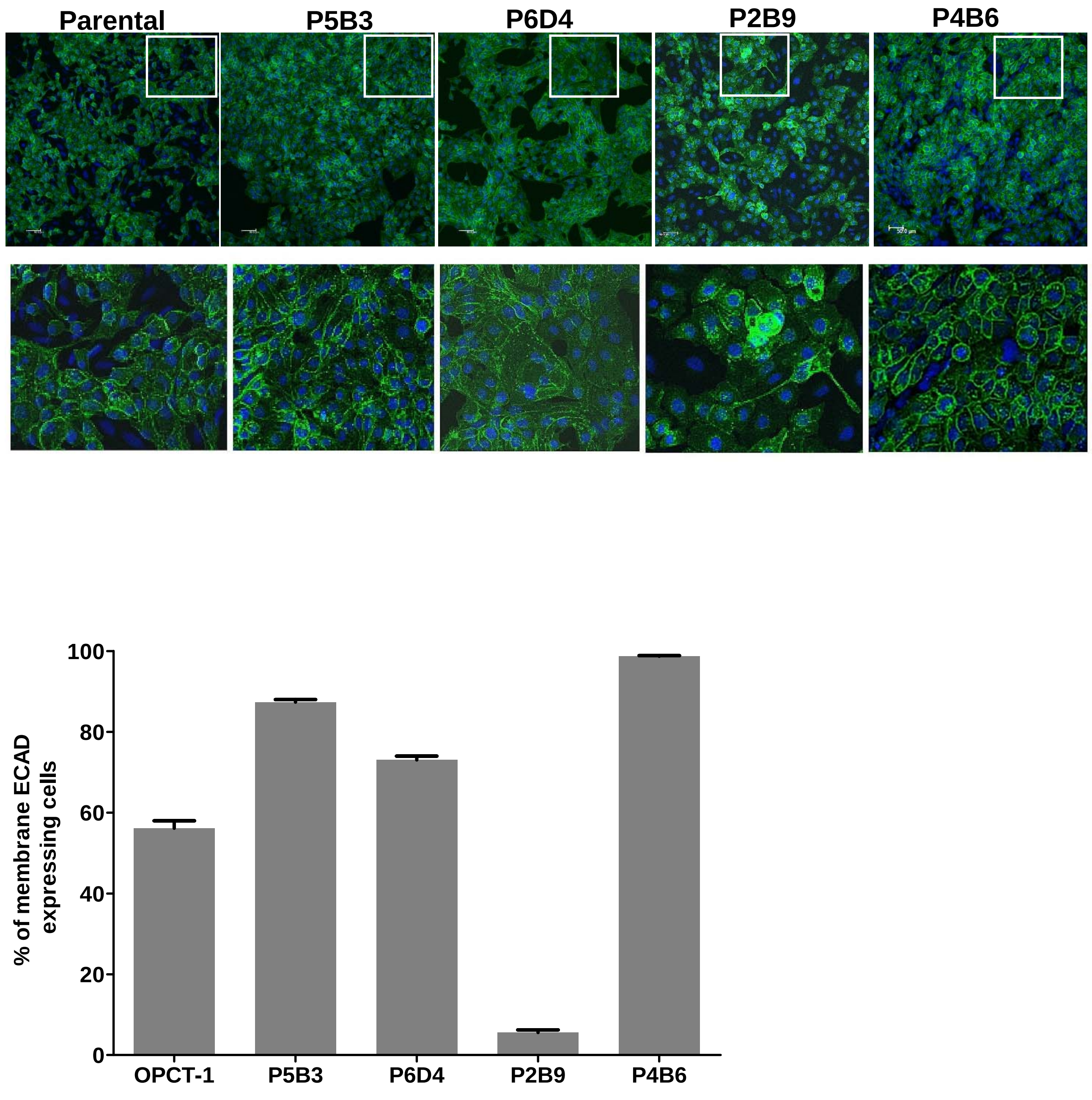

Supplementary Figure 3: Confocal images showing the localisation of E-cadherin in parental and clonal progenies of OPCT-1 and bar graph showing percentage of membrane localised E-cadherin for each clone and the parental line.(magnified images of selected regions also provided for better visualisation of E-cadherin localisation. Scale bar=50µM.

Supplementary Figure 4

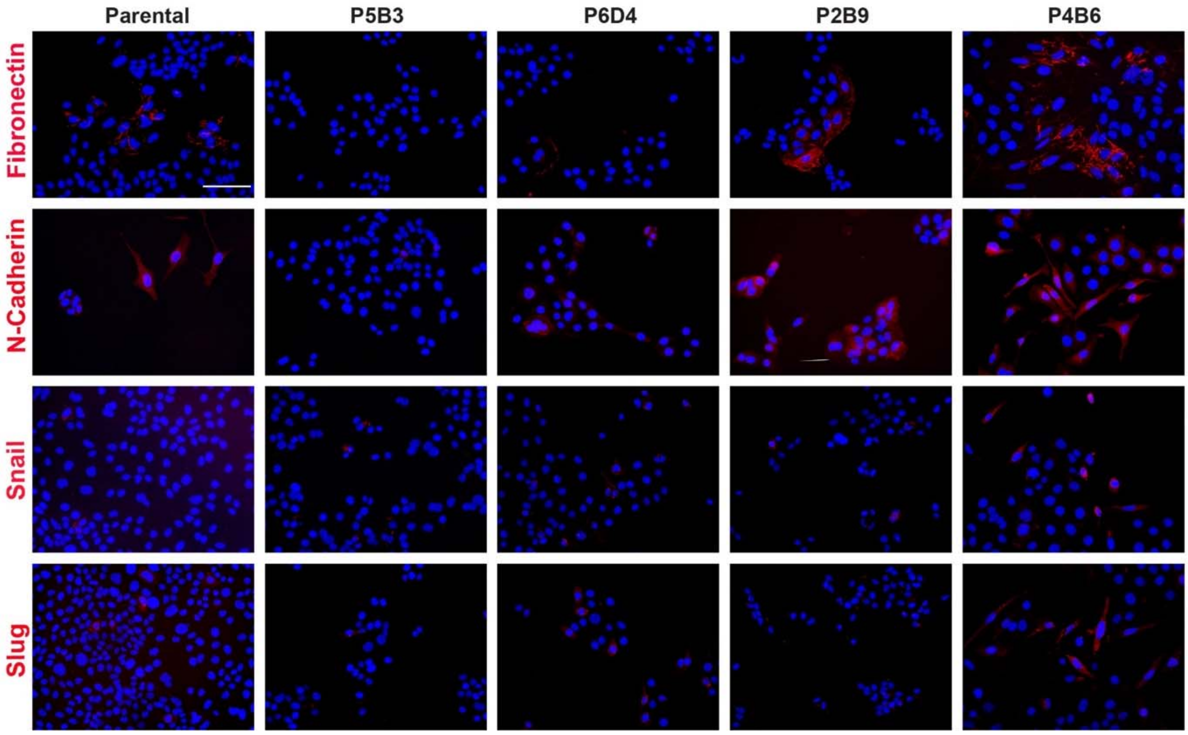

Supplementary Figure 4: Immunofluorescence of fibronectin, N-Cadherin, Slug and Snail in the parental OPCT-1 and the four clones. Magnification 20x; scale bar 50 μm.

Supplementary Figure 5

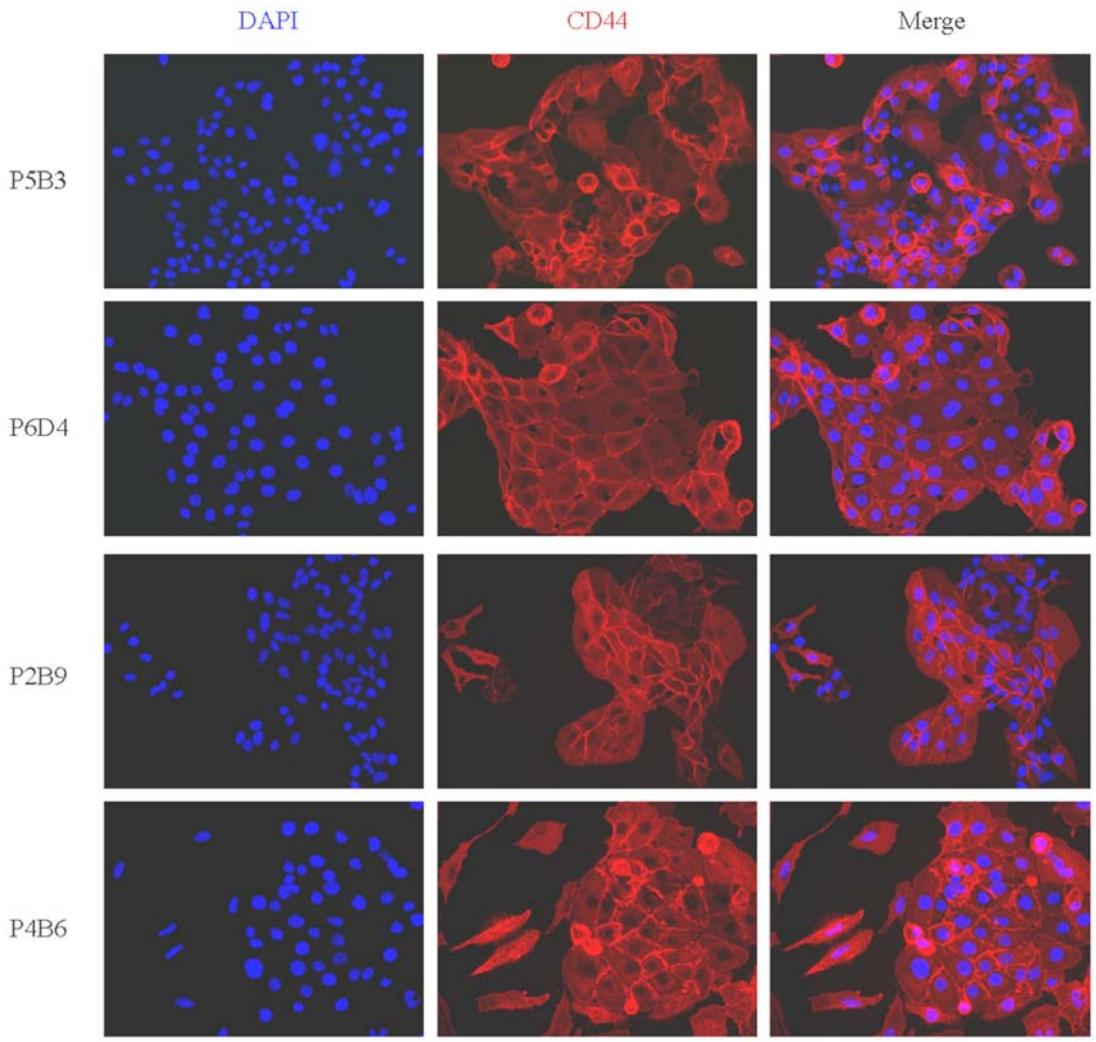

Supplementary Figure 5: *Immunofluorescent staining of clones P5B3, P6D4, P5F3, P2B9, and P4B6 using a mouse monoclonal antibody against CD44. Secondary antibody labelling was achieved using an anti-mouse AlexaFluor®568-conjugated secondary antibody and nuclear staining was performed using mounting media with DAPI (n=3). Representative images. Image magnification at x20.*

Supplementary Figure 6

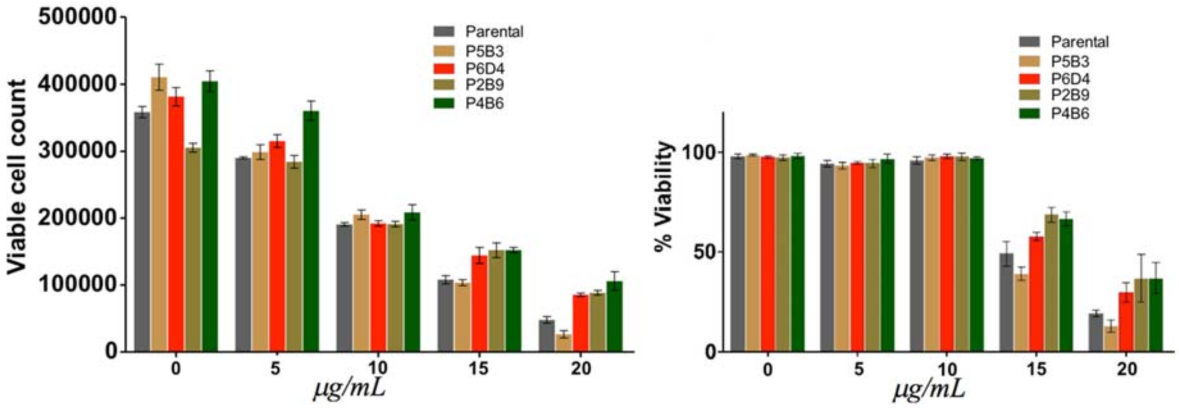

Supplementary Figure 6: *Optimal concentration of mitomycin for wound healing assay was determined using cultured cells (1.5x10^5 cells/well seeded overnight in a 24 well plate). the cells were treated with different concentrations of mitomycin for 2 h. after two hours the cells were washed three times with sterile PBS and the wells were replenished with normal tissue culture media. after 24 h in normal growth conditions the cells viability and number had been determined using an automated cell counter (NucleoCounter® NC-250™). 10 ug/mL of mitomycin for 2 h was found to be optimal for arresting cell proliferation without compromising viability.*

Supplementary Figure 7

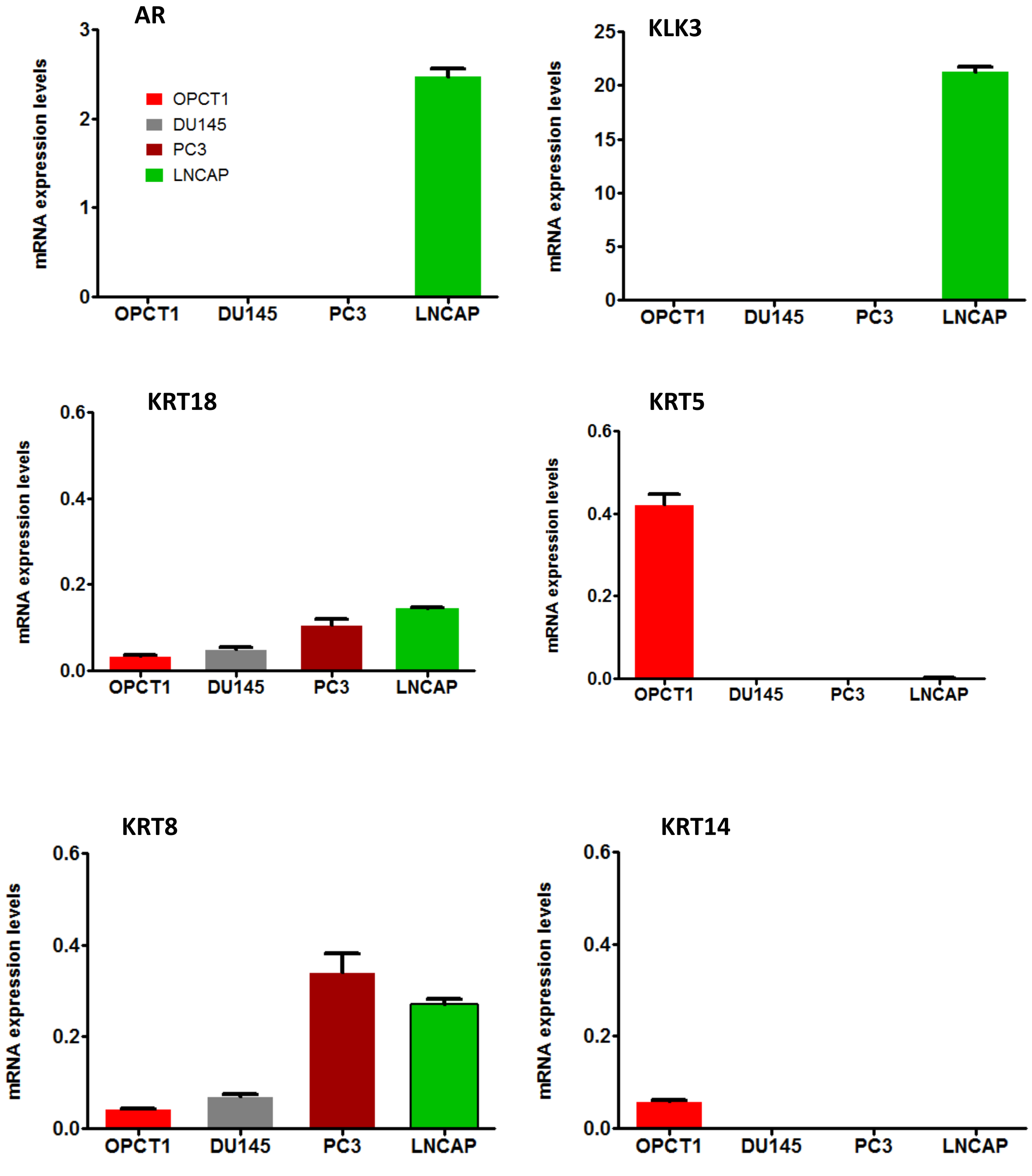

Supplementary Figure 7: *Expression of different markers used to differentiate basal, intermediate and luminal characteristics of prostate cancer cells using qRT PCR. Along with OPCT-1, other classical cell lines (LNCAP, DU145 and PC3) were also profiled. As is known from the literature, LNCAP showed a prominent luminal phenotype with the expression of androgen receptor (AR), PSA (KLK3) and cytokeratin (CK)-18 and 8, with CK-5 and CK14 being completely absent. Similar to DU145 and PC3, OPCT-1 lack the expression of AR and PSA, thereby indicating their non-luminal characteristics. Although high expression of CK-5 and low expression of CK14 was uniquely detected in OPCT-1, expression of a key the basal phenotype marker, p63, was not detected, thereby indicating a transit amplifying genotype in OPCT1.*

Supplementary Figure 8

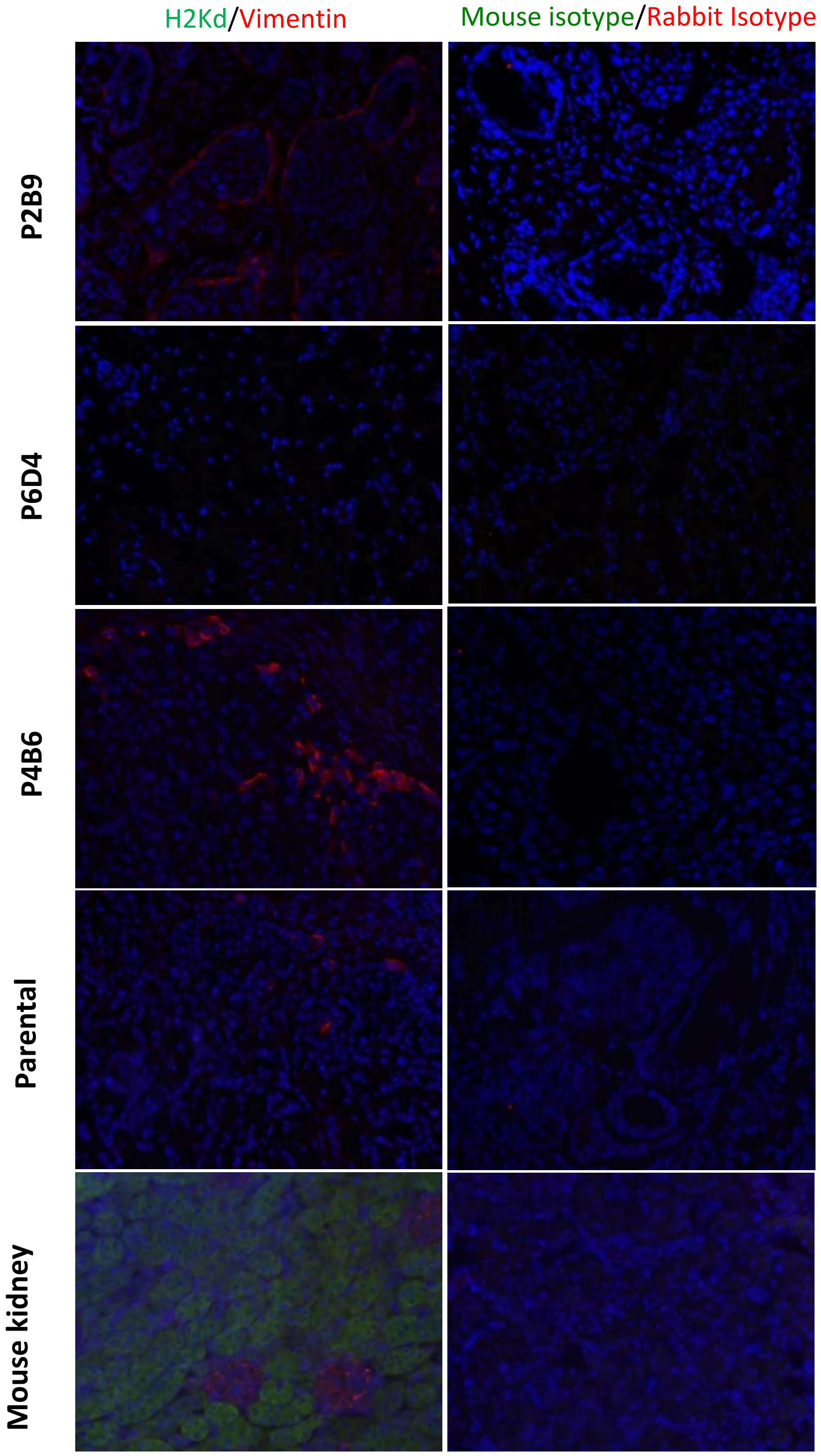

Supplementary Figure 8: *Representative immunofluorescent staining of tumour sections derived from clones P5B3, P6D4, P2B9, P4B6 and parental OPCT-1 and murine kidney using a monoclonal antibody specific for murine MHC class I H-2^{Kd}. Sections were labelled with a murine monoclonal antibody against MHC class I H2^{Kd} and a rabbit polyclonal antibody against murine vimentin. Primary antibody staining was detected using anti-mouse Alexa Fluor® 488 and anti-rabbit Alexa Fluor® 568 antibodies (x20 magnification). The absence of H2^{Kd} staining in vimentin positive cells indicates that these cells are of human origin.*

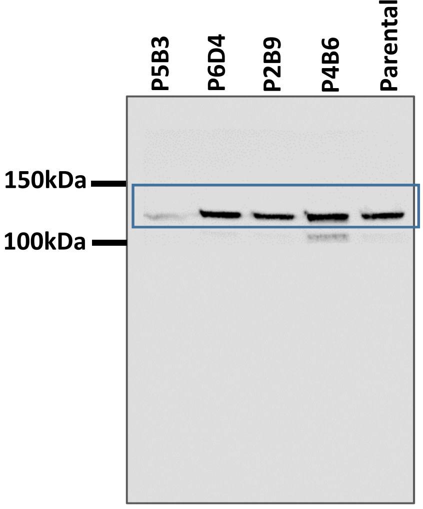

N-Cadherin

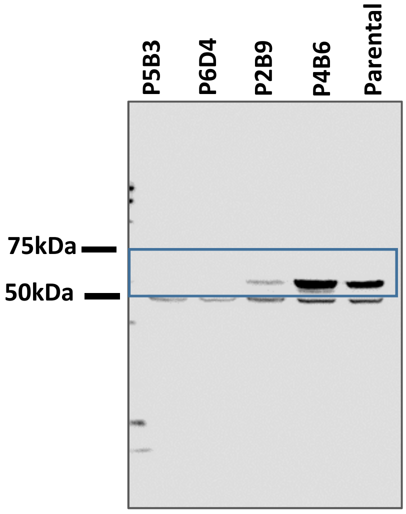

Vimentin

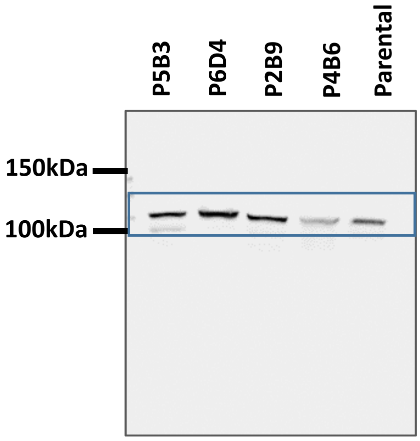

E-cadherin

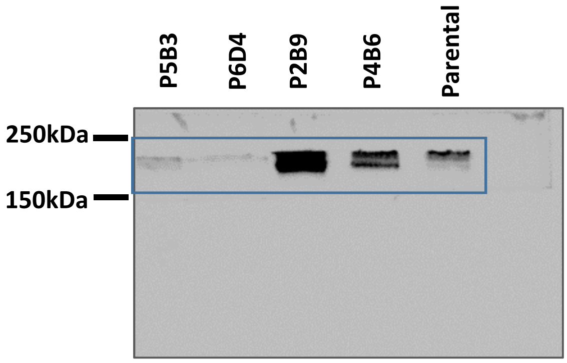

Fibronectin

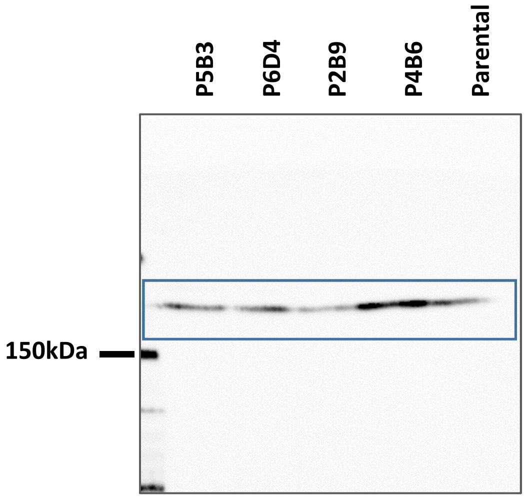

ZEB1

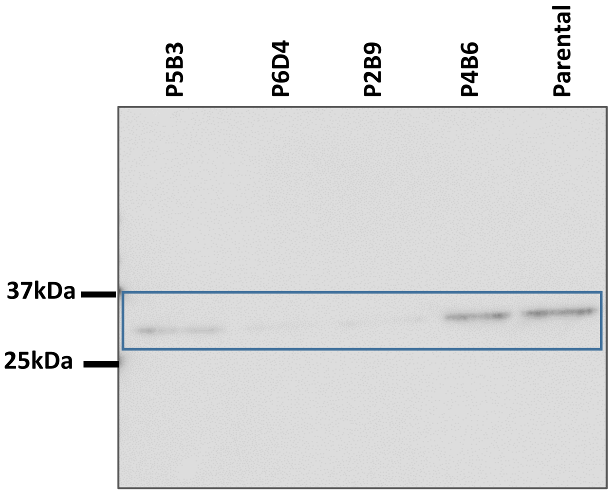

SNAIL

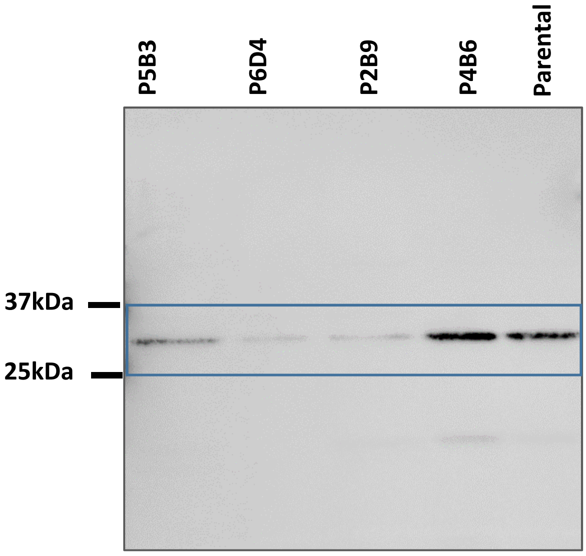

SLUG

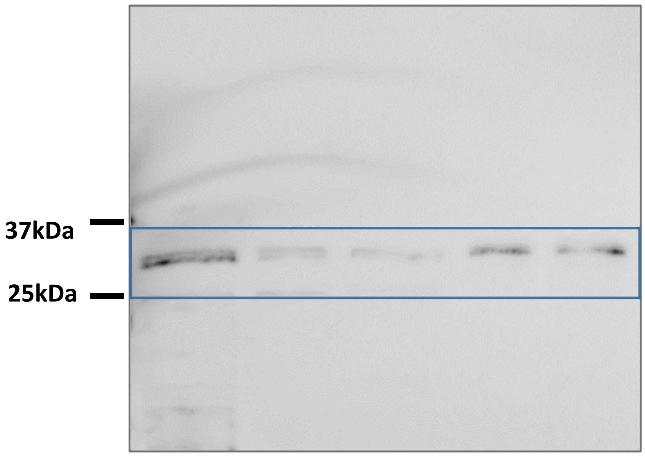

SOX2

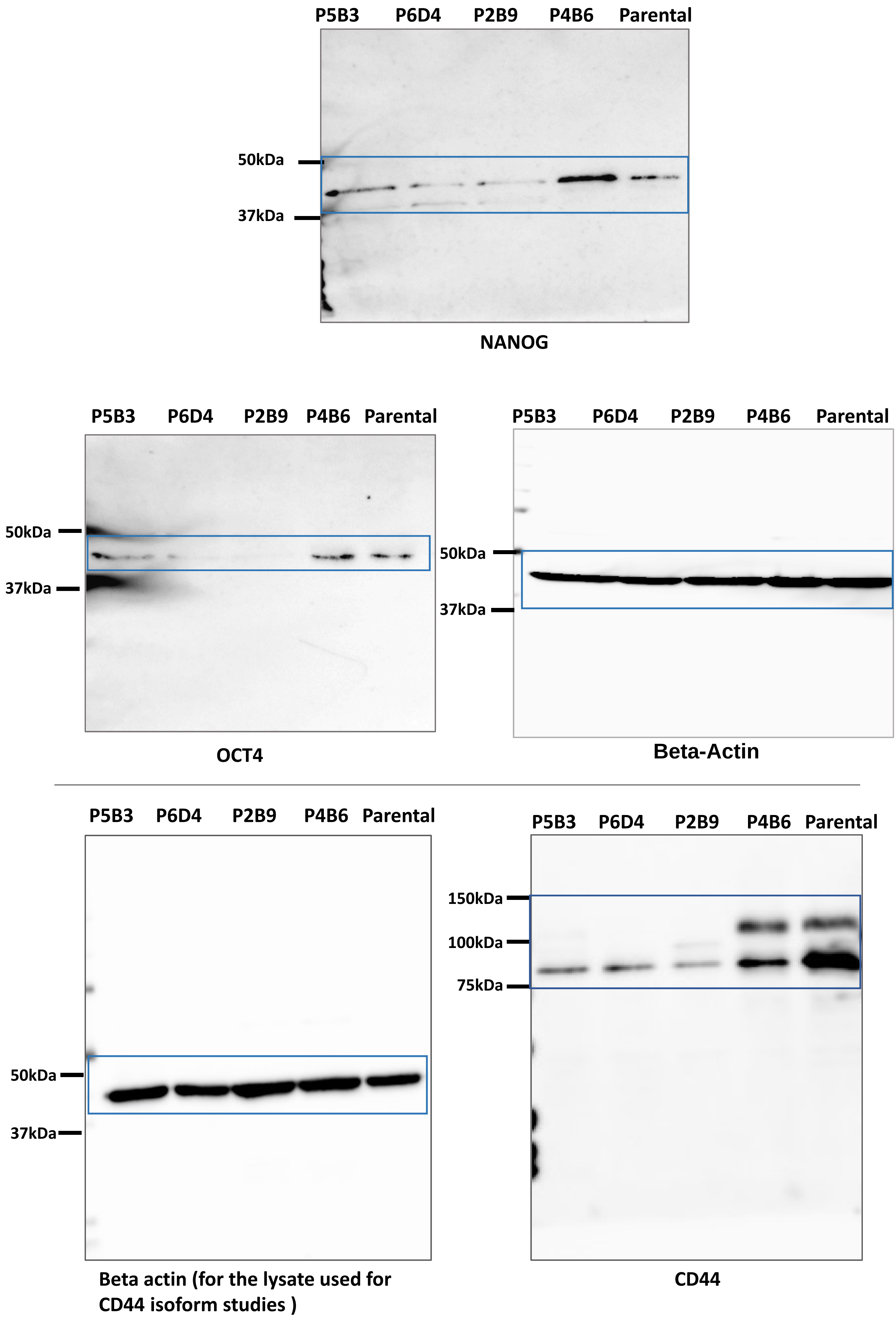

Supplementary Figure 9: Unprocessed original scans of Western blots

Supplementary Table 1: Demonstrating the results reporting the expression of E-Cadherin, Vimentin and CD44 by 51 OPCT-1 clones screened by immunofluorescence

Clone	*E-Cadherin*	*Vimentin*	*Cellular co-expression*	*CD44*
P1C7	+++	+++	*Yes*	+++
P1D2	+++	++	*Yes*	+++
P1D4	+++	++	*Yes*	+++
P1E6	+++	+++	*Yes*	+++
P1G3	+++	++	*Yes*	+++
P2B9	+++	+++	*Yes*	+++
P2D8	+++	++	*Yes*	+++
P2E7	+++	+	*Yes*	+++
P2E9	+++	+	*Yes*	+++
P2F3	+++	+	*Yes*	+++
P2G3	+++	+	*Yes*	+++
P3B11	+++	+	*Yes*	+++
P3D3	+++	+	*Yes*	+++
P3D10	+++	+	*No*	+++
P3D11	+++	++	*Yes*	+++
P3E5	+++	-	*n/a*	+++
P3G3	+++	++	*Yes*	+++
P3G10	+++	++	*Yes*	+++
P3H8	+++	+++	*Yes*	+++
P4B6	+++	+++	*Yes*	+++
P4C3	+++	+	*Yes*	+++
P4C7	+++	+	*Yes*	+++
P4E10	+++	+	*Yes*	+++
P4F2	+++	+	*Yes*	+++
P4F4	+++	+++	*Yes*	+++
P4G6	+++	+++	*Yes*	+++
P4G10	+++	++	*Yes*	+++
P5A5	+++	+	*Yes*	+++
P5A6	+++	+	*Yes*	+++
P5B3	+++	-	*n/a*	++
P5B7	+++	+++	*Yes*	+++
P5C3	+++	+	*Yes*	+++
P5D4	+++	+++	*Yes*	+++
P5D6	+++	+	*No*	+++
P5E4	+++	+	*Yes*	+++
P5E8	+++	+	*Yes*	+++
P5F8	+++	+++	*Yes*	+++
P5H8	+++	++	*Yes*	+++
P5H9	+++	++	*Yes*	+++

Clone	*E-Cadherin*	*Vimentin*	*Cellular Co-expression*	*CD44*
P5F2	+++	+++	*Yes*	+++
P5F3	+++	++	*Yes*	+++
P6D4	+++	+	*Yes*	+++
P6E6	+++	+++	*Yes*	+++
P6H5	+++	++	*Yes*	+++
P6C7	+++	++	*Yes*	+++
P6D1	+++	++	*Yes*	+++
P6D2	+++	+++	*Yes*	+++
P6E10	+++	++	*Yes*	+++
P6F12	+++	+	*No*	+++
P5A2	+++	++	*Yes*	+++
P5G11	+++	+	*Yes*	+++

+++ expressed by ~ 10-100% of cells ++ expressed by ~ 1-10% of cells + expressed by ~ <1% of cells – no positive cells observed
Yes = individual cells expressing both markers present No = only single-positive cells observed

Supplementary Table 2: List of primers used for the qRT PCR characterisation

Primer name	Sequence (5'-3')
**CDH1-F**	TGCCCAGAAAATGAAAAAGG
**CDH1-R**	GTGTATGTGGCAATGCGTTC
**CDH2-F**	ACAGTGGCCACCTACAAAGG
**CDH2-R**	CCGAGATGGGGTTGATAATG
**FN1-F**	CAGTGGGAGACCTCGAGAAG
**FN1-R**	TCCCTCGGAACATCAGAAAC
**VIM-F**	GAGAACTTTGCCGTTGAAGC
**VIM-R**	GCTTCCTGTAGGTGGCAATC
**SNAI1-F**	CCTCCCTGTCAGATGAGGAC
**SNAI1-R**	CCAGGCTGAGGTATTCCTTG
**TWIST-F**	GGAGTCCGCAGTCTTACGAG
**TWIST-R**	TCTGGAGGACCTGGTAGAGG
**SNAI2-F**	GGGGAGAAGCCTTTTTCTTG
**SNAI2-R**	TCCTCATGTTTGTGCAGGAG
**FOXC2-F**	GCCTAAGGACCTGGTGAAGC
**FOXC2-R**	TTGACGAAGCACTCGTTGAG
**ZEB1- F**	GGCATACACCTACTCAACTACGG
**ZEB1- R**	TGGGCGGTGTAGAATCAGAGTC
**OCT4- F**	GATCACCCTGGGATATACAC
**OCT4- R**	GCTTTGCATATCTCCTGAAG
**SOX2-F**	ATAATAACAATCATCGGCGG
**SOX2-R**	AAAAAGAGAGAGGCAAACTG
**NANOG- F**	CCAGAACCAGAGAATGAAATC
**NANOG- R**	TGGTGGTAGGAAGAGTAAAG
**HPRT-F**	TGACACTGGCAAAACAATGCA
**HPRT-R**	GGTCCTTTTCACCAGCAAGCT
